# Metal tolerance and biosorption capacities of bacterial strains isolated from an urban watershed

**DOI:** 10.3389/fmicb.2023.1278886

**Published:** 2023-10-23

**Authors:** Grace Pagnucco, Dustin Overfield, Yanesa Chamlee, Claudia Shuler, Amin Kassem, Somie Opara, Hawraa Najaf, Lana Abbas, Oliver Coutinho, Aleksa Fortuna, Fatima Sulaiman, James Farinas, Reis Schittenhelm, Brian Catalfano, Xiaohua Li, Sonia M. Tiquia-Arashiro

**Affiliations:** Department of Natural Sciences, University of Michigan-Dearborn, Dearborn, MI, United States

**Keywords:** biosorption, metal-resistant microbes, metal selectivity, exopolysaccharide-producing bacteria, heavy metal removal, Fourier-transform infrared analysis, scanning electron microscopy, transmission electron microscopy

## Abstract

Rapid industrialization and urbanization have led to widespread metal contamination in aquatic ecosystems. This study explores the metal tolerance and biosorption characteristics of four bacterial strains (*Serratia* sp. L2, *Raoultella* sp. L30, *Klebsiella* sp. R3, and *Klebsiella* sp. R19) isolated from Saint Clair River sediments. These strains effectively removed various metal cations (As^3+^, Pb^2+^, Cu^2+^, Mn^2+^, Zn^2+^, Cd^2+^, Cr^6+^, and Ni^2+^) in single and multi-metal solutions. Minimum inhibitory concentration (MIC) assays revealed strain-specific variations in metal tolerance, with L2 and L30 exhibiting higher tolerance. Surprisingly, R3 and R19, despite lower tolerance, demonstrated superior metal removal efficiency, challenging the notion that tolerance dictates removal efficacy. In single-metal solutions, R3 and R19 excelled at extracting various metal ions, while competitive binding in multi-metal solutions hindered removal. However, R3 and R19 retained higher removal efficiencies, possibly due to enhanced flocculation activities facilitating metal-ion contact. Comprehensive Fourier-transform infrared (FTIR) analysis highlighted the strains’ metal-binding capabilities, with novel peaks emerging after metal exposure, indicative of extracellular polymeric substance (EPS) production. Scanning electron microscopy (SEM) and energy-dispersive X-ray spectroscopy (EDX) confirmed metal accumulation on bacterial surfaces and within cytoplasmic regions and revealed morphological changes and metal adsorption patterns, emphasizing the strains’ ability to adapt to metal stress. Scanning transmission microscopy (STEM) and EDX analysis uncovered metal accumulation within bacterial cells, underscoring the complexity of microbial-metal interactions. This study also confirms that the simultaneous presence of an aqueous solution may cause a mutual inhibition in the adsorption of each metal to the EPS resulting in reduced metal uptake, which emphasizes the need to select specific bacterial strains for a given metal-containing effluent. The differences in metal distribution patterns between *Klebsiella* sp. R19 and *Raoultella* sp. L30 suggest species-specific metal accumulation strategies driven by environmental conditions and metal availability. The heavy metal-removing capabilities and the ability to grow over a wide range of metal concentrations of the strains used in this study may offer an advantage to employ these organisms for metal remediation in bioreactors or *in situ*.

## 1. Introduction

Wastewater contamination has emerged as a critical environmental concern in recent years. The escalating processes of industrialization and urbanization have contributed to the significant release of heavy metals into aquatic ecosystems (Boening, 2000; Zamora-Ledezma et al., 2021; Goswami and Neog, 2023). The release of heavy metals into aquatic environments arises from a variety of sources, including industrial discharges, urban runoff, and agricultural activities (Tiquia, 2010; Oest et al., 2018; Patel et al., 2019; Narwal et al., 2023). These pollutants pose a substantial threat to both aquatic ecosystems and human health due to their persistence and potential toxicity (Goswami and Neog, 2023). As heavy metals accumulate in aquatic environments, they can disrupt the delicate balance of these ecosystems, causing harm to aquatic life and endangering the health of humans who rely on these water bodies for various purposes, including drinking water and recreational activities (Tiquia, 2011; Sharma et al., 2023). Additionally, heavy metal contamination can have far-reaching ecological implications, affecting the food chain and ultimately impacting terrestrial ecosystems as well (Ledin, 2000; Ahmad et al., 2021). Among the heavy metals under rigorous investigation are chromium (Cr), cadmium (Cd), copper (Cu), zinc (Zn), mercury (Hg), lead (Pb), nickel (Ni), manganese (Mn), and arsenic (As), primarily due to their pronounced threats to both public health and the environment (Boulanger and Nikolaidis, 2003; Tchounwou et al., 2012; Queiroz et al., 2021). These heavy metals find their way into the environment through human-driven activities like mining and agriculture (Goswami and Neog, 2023). Traditional techniques such as reverse osmosis, ion exchange, precipitation, and solvent extraction have been utilized to treat wastewater before discharge into natural ecosystems. However, these methods either incur substantial costs or fall short in terms of efficacy (Volesky, 1990; Qasem et al., 2021). In response, the employment of microorganisms for heavy metal removal has emerged as a highly promising alternative. This approach holds advantages by preventing secondary pollution and offering cost-effectiveness through the avoidance of sludge disposal requirements (Kim et al., 1996; Bruins et al., 2000; Lakherwal, 2014).

Bioremediation processes using microorganisms are cost effective and are highly efficient as compared to physicochemical methods mentioned above; therefore, over the last several decades attention has focused toward exploiting microbes for heavy metal bioremediation (Tiquia-Arashiro, 2018). The bioremediation of heavy metals can be categorized into two main approaches: bioaccumulation, which is an actively controlled process necessitating the uptake of heavy metal ions by living biomass, and biosorption, a passive process where metal cations are adhered to non-living biomass (Razzak et al., 2022; Jeyakumari et al., 2023). In the context of bioaccumulation, energy is expended for the absorption of metal ions, usually achieved through interactions with the cell wall. Following this, intracellular uptake takes place, with metal ions permeating the cell membrane and binding to active sites provided by polysaccharides and proteins. In contrast, biosorption capitalizes on the presence of functional groups in the cell wall or exported metabolites within the external environment. The mechanisms behind biosorption include ion exchange, complexation, precipitation, reduction, and chelation (Vishan et al., 2017; Priya et al., 2022; Sreedevi et al., 2022).

Accordingly, understanding the mechanisms by which microorganisms sequester toxic heavy metals is crucial to the development of microbial processes that will concentrate, remove, and recover metals from industrial effluents. The bioavailability of metals in the environment is an important factor for metal toxicity since soluble metals can readily penetrate cell membranes (Roane, 1999; Tiquia-Arashiro, 2018; Jeong et al., 2023). To counteract this, bacteria employ immobilization strategies to counteract the toxic effect of heavy metals, which includes precipitation, intracellular accumulation, and extracellular sequestration in exopolysaccharides (EPSs) (Higham and Sadler, 1984; Roane, 1999; van Hullebusch et al., 2003; Tiquia-Arashiro, 2018; Vandana et al., 2023). EPSs typically consist of polysaccharides featuring ionizable functional groups that can bind to anionic species. This transformation facilitates electrostatic interactions with cationic metal ions, ultimately leading to the immobilization of heavy metals within the EPS matrix (Saba et al., 2019). The effectiveness of EPS-mediated biosorption in efficiently sequestering toxic metals has led to its extensive investigation (Shameer, 2016; Vishan et al., 2017; Saba et al., 2019; Sharma and Saraf, 2023). As a result, biosorption via EPS has garnered substantial research attention. Heavy metals do not only need to be remediated from the ecosystem but are also required to be recovered from every possible source given their importance in commercial and industrial applications. The interaction between several metal cations and the EPS-producing microorganisms combined with their potential for removing heavy metals from polluted waters has stimulated scientific interests due to their ecological importance and practical implications.

Despite the isolation of diverse bacterial strains, the significance of isolating and characterizing indigenous bacteria remains paramount. This approach is not only environmentally sound but also preserves the ecological balance within their specific habitats. This assertion is corroborated by studies that have harnessed metal-tolerant bacteria sourced from industrial effluents (Mishra et al., 2013; Bhatt et al., 2023). In our previous study (Bowman et al., 2018), we isolated Pb-resistant bacterial strains, *Klebsiella* sp. R3, *Klebsiella* sp. R19, *Serratia* sp. L2, and *Raoultella* sp. L30 from sediments of the Saint Clair River. These bacterial strains remove large amounts of Pb^2+^ from solution and produce a high rate of flocculation activity (Bowman et al., 2018). Furthermore, these bacterial strains are well adapted to unfavorable conditions due to their resistance to metals (e.g., Pb) and antibiotics and can grow in a wide range of temperatures. These characteristics may help in developing an effective process for wastewater treatment. However, the metal removal of these strains in aqueous multi-metal solutions has not been investigated in detail, which is particularly useful for building up processes aimed at recovering metals from industrial wastewaters. This study aims to (1) assess the selective metal removal abilities of the bacterial strains in the presence of multiple heavy metals (As^3+^, Pb^2+^, Cu^2+^, Mn^2+^, Zn^2+^, Cd^2+^, Cr^6+^), (2) investigate the interactions between these metals during the sorption process, and (3) uncover mechanisms of the biosorption process using FT-IR, SEM–EDX, and STEM-EDX techniques.

## 2. Materials and methods

### 2.1. Bacterial strains

In this study, the strains were grown on M9 minimal media (M9 salts [BD Difco, Franklin Lakes, NJ], 20% glucose, 1 M MgSO_4_, 1 M CaCl_2_). The biochemical properties of bacterial strains were characterized using the API 20E system (Bio-Mérieux, Marcy-l’Étoile, France). Furthermore, the 16S rRNA sequence of the bacterial strains were determined by direct sequencing of the PCR product. DNA extracts from each strain were prepared using the DNEasy kit (Qiagen, Inc., Valencia, CA). Genomic DNA from these isolates were extracted using the DNEasy kit. Identification of isolates was performed by amplifying the full-length 16S rRNA genes using bacteria-specific primers sequence FD1 (5′ AGA GTT TGA TCC TGG CTC AG 3′) and 1540r (5′ > AAG GAG GTG ATC CAG CC < 3′) (Weisburg et al., 1991) with cycling conditions described previously (Tiquia et al., 2007, 2008; Cho et al., 2012; Nguyen et al., 2013; Plecha et al., 2013). The purified PCR products were sequenced and analyzed directly without cloning. Sequence fragments generated from a given template were edited against electropherograms and then assembled into contigs using SeqMan (Lasergene DNASTAR, Inc., Madison, WI). Two to four overlapping fragments (from both coding and noncoding strand) were used to assemble the contigs. Chimeric sequences were checked by the Check_Chimera program available at the Ribosomal Database Project (RDP II) (Hugenholtz and Huber, 2003). Complete 16S rRNA gene sequences were compared with other reference sequences as available in the NCBI database using the Basic Local Alignment Search Tool (BLAST) algorithm. Closely related sequences were retrieved from Genbank and were aligned along with the 16S rRNA gene sequences of the bacterial strains using the ClustalW program. Phylogenetic trees (Supplementary Figure S1) were constructed from the evolutionary distance matrix calculated through the neighbor-joining method (Saitou and Nei, 1987). Neighbor joining analysis was performed using MEGA 11 (Tamura et al., 2021). Based on the morphological, physiological, biochemical characteristics (Supplementary Table S1) and comparative analysis of the sequence to references retrieved from the NCBI database, the strains were found to be similar to the 16S rRNA sequences of *Klebsiella* sp. (strain R2), *Klebsiella* sp. (strain R19), *Serratia* sp. (strain L2) and *Raoultella* sp. (strain L30).

### 2.2. Determination of minimum inhibitory concentrations to metals

The bacterial strains used in this study are resistant to Pb and grew in enrichment cultures containing high concentrations (1.25 or 1.5 g L^−1^) of Pb (NO_3_)_2_ (Bowman et al., 2018). To determine their resistance to other metals (As^3+^, Pb^2+^, Cu^2+^, Mn^2+^, Zn^2+^, Cd, ^2+^ Cr^6+^, or Ni^2+^), the MIC on the strains to these metals was carried out. Stock solutions of As (NaAsO_2_), Cr (K_2_Cr_2_O_7_), Cd (CdCl_2_), Cu (CuCl_2_.2H_2_0), Pb (Pb [NO_3_]_2_), Mn (MnCl_2_.4H_2_O), Ni (NiCl_2_.6H_2_O), and Zn (ZnSO_4_.7H_2_O) were prepared to achieve the final concentration of 1,000 and 10,000 mg L^−1^ with distilled water. The metal solutions were filter sterilized using 0.2 μm Nalgene vacuum filtration system (Thermo Scientific, Waltham, MA). Polycarbonate filters were required for the Cu study. For other metals, polysulfone filters were used to prevent sorption of metals to the filter apparatus (Shuttleworth and Unz, 1993; Wu et al., 2005). Susceptibility testing of the strains to eight different metals was conducted in M9 minimal media amended with metals. Metal-amended media were prepared by adding increasing amounts of metal stock solutions to the autoclaved media. The broth media (200 μL) were poured into 96-well plates and inoculated with mid-logarithmic-phase cultures (20 μL). The plates were incubated at room temperature with shaking (SBT1500 Microplate Shaker, Southwest Science, Hamilton, NJ) at 150 rpm. The MICs (minimal inhibitory concentrations) reported in this study were the lowest concentration of the metal that inhibited bacterial growth after an incubation of 24 h at room temperature in the dark. Growth media inoculated with bacterial strains without metals were used as positive controls. Growth of the strains was monitored by optical density at 595 nm with Sunrise microwell plate reader (Tecan, Research Triangle Park, NC). The absence of growth was determined by the detection threshold of the plate reader.

### 2.3. Metal removal assay in aqueous solutions

Cells of each culture (100 mL) were confined in dialysis tubing (12–14 kDa of molecular weight cut-off; Spektra/por 5, Spectrum Laboratories, CA) and pretreated with 0.1 N HCl to remove the metal ions possibly bound to the negatively charged groups of the cell envelopes. After 30 min of soaking in 0.1 N HCl, the cell cultures were dialyzed against deionized water for 24 h with continuous stirring at 100 rpm, to remove residual HCl from the treated cultures. In the mono-metal systems, dialyzed cultures were subsequently transferred in 1 L aqueous solutions containing 10 mg L^−1^ of As^3+^, Pb^2+^, Cu^2+^, Mn^2+^, Zn^2+^, Cd^2+^, Cr^6+^, or Ni^2+^ for 24 h. This metal concentration (10 mg L^−1^) is like that used in other dialysis assays to investigate the binding capacity of bacterial cells or bacterial EPS with metals (Perez et al., 2008; Pereira et al., 2011; Wang et al., 2014; Bowman et al., 2018). To avoid any possible hydroxide precipitation, the pH of the system (biosorbent + metal solution) was adjusted to 4.5–5.5 by addition of either 1 N HCl or 1 N NaOH. The experiment was performed at room temperature. For the multi-metal systems, 10 mg L^−1^ of each metal was used. Such concentration was used for each metal to be consistent with the concentrations used for the single-metal assays.

Ten mL of the aqueous solutions were withdrawn at different time intervals over 24 h and filtered through 0.2 μm Acrodisc syringe filters (Sigma-Aldrich, Saint Louis, MO). The metal uptake by the bacterial strains was determined from the difference between the concentration of the metals in solution using an atomic absorption spectrometer (PinAAcle 900 T, Perkin Elmer, MA). The As^3+^, Pb^2+^, Cu^2+^, Mn^2+^, Zn^2+^, Cd^2+^, Cr^6+^, and Ni^2+^ were determined at 193.70, 283.31, 324.75, 279.48, 213.86, 228.00, 357.87, and 232.0 nm, respectively. The amount of metal uptake in blanks (carried out in parallel with just 100 mL of culture media without cells) was also monitored to confirm that negligible metal was lost from solution. The amount of metals removed in blanks, which ranged from 0.1 to 0.2 mg L^−1^, was subtracted from the experimental values obtained in the tests with the bacterial cultures.

Specific metal uptake (*q*) was expressed as mg metal removed per g of dry biomass. The dry weight (g L^−1^) was determined by centrifugation 10 mL of the dialyzed cultures, followed by drying at 50°C until a constant weight was reached (Volesky and May-Philips, 1995). The experiments were performed in triplicates and the data were reported as mean values ± standard deviation. As metal sorption was followed, cell viability was also determined at the beginning and at 24 h using the plate count method (Gerhardt et al., 1994).

### 2.4. FT-IR analysis of bacterial biomass

Fourier transform infrared spectroscopy (FT-IR) was performed to identify the functional groups in the bacterial strains that might be involved in metal uptake during the biosorption process. This technique has proven to be effective in providing structural information on metal cation binding in microbes (Gupta et al., 2020). FT-IR analysis was carried out on cells before and after metal uptake in aqueous solution containing the 8 metals (As^3+^, Pb^2+^, Cu^2+^, Mn^2+^, Zn^2+^, Cd^2+^, Cr^6+^, and Ni^2+^), each had a concentration of 10 mg/L. The infrared spectra of the control before and after metal uptake were analyzed on lyophilized cells using Attenuated Total Reflection (ATR).

### 2.5. SEM–EDX analysis

Field emission scanning electron microscopy (Tescan Mira3 FEG SEM, Tescan USA, Inc., Pleasanton, CA 94588, United States) coupled with energy dispersive X-ray spectroscopy was carried out to examine the outer morphology of the bacterial cells (Raoultella sp. L30 and *Klebsiella* R19) grown without metal stress and bacterial cells grown with metals. Samples were fixed in 2.5% glutaraldehyde in Sorensen phosphate buffer (pH 7.2), dehydrated with increasing solutions of ethanol (30, 50, 70, 80, 90, 95, and 100%) and HDMS. Prepared samples were placed on the sample holder (stub) with carbon tape. To improve electron conduction, the samples were sputter coated with carbon.

### 2.6. STEM-EDX analysis

Scanning Transmission electron microscopy with energy dispersive X-ray (EDS) analysis (Thermo Fisher Talos F200X G2, Waltham, Massachusetts, United States) was used to determine the location of the metals in the cell. Strains R19 and L30 were washed with phosphate buffer saline (pH 6.8). After washing, the samples were embedded in agarose and then fixed in 3% Glutaraldehyde +3% paraformaldehyde solutions in 0.1 M Cacodylate buffer (pH 7.2). After 24 h, the cells were fixed with osmium tetroxide (2%) and potassium ferrocyanide (1%) 0.1 M carbonate buffer for 1 h and then washed with 0.1 M carbonate buffer and 0.1 M Na_2_ + acetate buffer (pH 5.2) before en-bloc staining for 1 h. Using an automated tissue processor (Leica EM TP, Leica Biosystems, Deerfield, Illinois, USA) the samples were washed with 0.1 M carbonate buffer and 0.1 M Na_2_ + acetate buffer (pH 5.2) and then dehydrated with increasing concentrations of ethanol before infiltrated with acetone and Spurr’s resin. Thin sections of 60–80 nm thick were obtained using an ultramicrotome (Leica EM UC7, Leica Biosystems, Deerfield, Illinois, United States) and stained with uranyl acetate. The section was fixed on a carbon grid and examined TEM.

### 2.7. Nucleotide sequence accession numbers and taxon numbers

The nucleotide sequence coding for 16S rRNA genes of *Klebsiella* sp. R3, *Klebsiella* sp. R19, *Serratia* sp. L2, and *Raoultella* sp. L30 were submitted to the GenBank database under the accession numbers OR143353, MG022653, OR143351, and OR143352, respectively. The respective digital protologue database (DPD) Taxon Number were: TA00308 (*Klebsiella* sp. R3), TA00308 (*Klebsiella* sp. R19), TA00309 (*Serratia* sp. L2), and TA00310 (*Raoultella* sp. L30).

## 3. Results

### 3.1. Tolerance to metals of the bacterial strains

Table 1 provides the minimum inhibitory concentrations (MICs) of the eight different metals for the respective bacterial strains. Overall, strains L2 and L30 (*Serratia* sp. strain L2 and *Raoultella* sp. strain L30) exhibited higher metal tolerance compared to strains R3 (*Klebsiella* sp.) and R19 (*Klebsiella* sp.). In a broader perspective, the strains demonstrated greater resilience against As^3+^ (ranging from 250 to 450 mg L^−1^), Pb^2+^ (ranging from 700 to 800 g mL^−1^), Mn^2+^ (>2,000 mg L^−1^), and Zn^2+^ (ranging from 500 to 1,100 mg L^−1^), in contrast to Cd^2+^ (ranging from 20 to 75 mg L^−1^), Cr^6+^ (ranging from 5 to 10 mg L^−1^), Cu^2+^ (ranging from 20 to 50 mg L^−1^), and Ni^2+^ (ranging from 50 to 75 mg L^−1^).

**Table 1:** Minimum inhibitory concentrations of metals.

Minimum inhibitory concentrations (MIC) (mg L^−1^)	Strains			
	*Klebsiella* sp. strain R3	*Klebsiella* sp. strain R19	*Serratia* sp. strain L2	*Raoultella* sp. strain L30
As	250	350	350	450
Cd	20	20	75	75
Cr	10	15	15	15
Cu	50	50	20	50
Pb	700	800	800	800
Mn	>2,000	>2,000	>2,000	>2,000
Ni	50	50	75	50
Zn	1,000	500	1,100	1,100

The hierarchy of metal tolerance for each bacterial strain was as follows: for *Klebsiella* sp. R3, it was Mn^2+^ > Zn^2+^ > Pb^2+^ > As^3+^ > Cu^2+^ = Ni^2+^ > Cd^2+^ > Cr^6+^; for *Klebsiella* sp. R19, it was Mn^2+^ > Pb^2+^ > Zn^2+^ > As^3+^ > Cu^2+^ = Ni^2+^ > Cd^2+^ > Cr^6+^; for *Serratia* sp. L2, it was Mn^2+^ > Zn^2+^ > Pb^2+^ > As^3+^ > Ni^2+^ = Cd^2+^ > Cu^2+^ > Cr^6+^; and for *Raoultella* sp. L30, it was Mn > Zn > Pb > As > Cd > Ni = Cu > Cr (Table 1).

### 3.2. Metal removal in single-metal and multi-metal systems

The investigation into the specific metal removal capabilities of the bacterial strains was conducted using individual single-metal solutions, each containing 10 mg L^−1^ of As^3+^, Pb^2+^, Cu^2+^, Mn^2+^, Zn^2+^, Cd^2+^, Cr^6+^, or Ni^2+^. The study spanned various time intervals over a 24-h period (as demonstrated in Supplementary Figure S2). Over time, the metals were gradually extracted from the solutions, with saturation of the metal removal capacity achieved within approximately 4 to 5 h (Supplementary Figure S2), except for As^3+^ (notably for strains L2 and L30) and Cr^6+^, for which minimal or negligible removal occurred. Generally, the metal removal efficiency was more pronounced for *Klebsiella* sp. R3 and *Klebsiella* sp. R19 (ranging from 4.4 to 318 mg g^−1^ dry mass) compared to *Serratia* sp. L2 and *Raoultella* sp. L30 (ranging from 0 to 155.42 mg g^−1^ dry mass) (Supplementary Figure S2).

**Figure 1:**
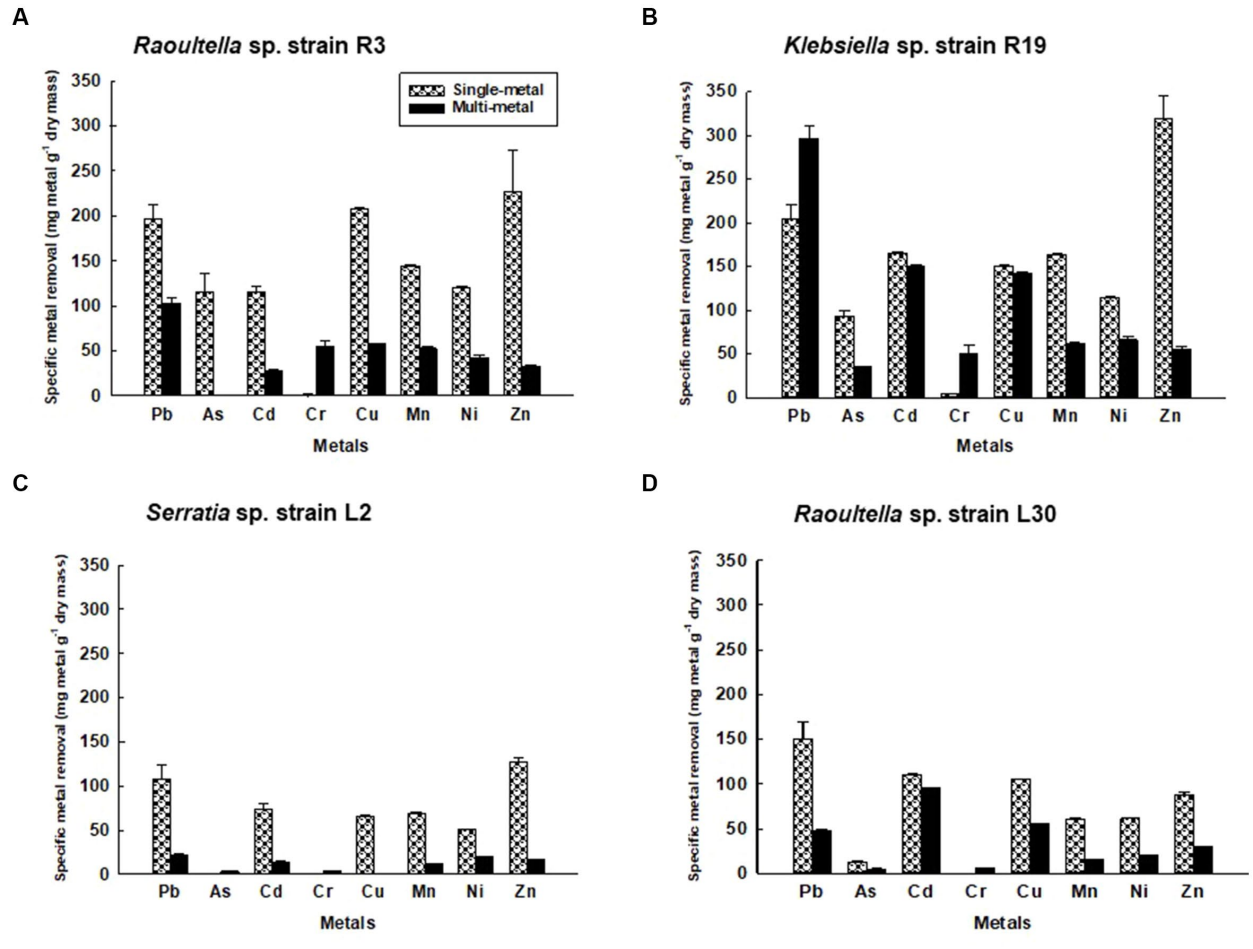
Metal removal by **(A)** *Klebsiella* sp. R3; **(B)** *Klebsiella* sp. R19; **(C)** *Serratia* sp. L2; and **(D)** *Raoultella* sp. L30 in single- (dotted bars) and multiple- (black bars) metal solutions. All the values are the mean of three replicates ± standard deviation.

Comparatively, the efficiency of metal removal in single-metal solutions was notably higher than that in mixed solutions containing all eight metal cations (as depicted in Figure 1). Notably, in single-metal solutions, *Klebsiella* sp. R3 exhibited removal efficiencies of ≥150 mg g^−1^ dry mass for Pb^2+^, Cu^2+^, and Zn^2+^ (Figure 1A). Similarly, *Klebsiella* sp. R19 displayed high removal efficiency for Pb^2+^, Cd^2+^, Cu^2+^, Mn^2+^, and Zn^2+^ (Figure 1B), while *Raoultella* sp. L30 showed effective removal of Pb^2+^ (Figure 1D). In the presence of all eight metal cations, the binding process was primarily inhibitory rather than stimulatory, as the cations competed for binding sites on the bacterial cells. Unexpectedly, a stimulatory effect on the removal efficiency of Pb^2+^ and Cr^6+^ was observed in strain R19 (Figure 1B) in the presence of multiple metal cations. Specifically, Pb^2+^ removal in the multi-metal solution was 31% higher compared to the solution with only Pb^2+^, and 91% higher compared to the solution with only Cr^6+^. A lesser stimulatory effect was observed for Cr^6+^ removal in strains L2 and L30 (Figures 1C,D).

Notably, the kinetics of metal uptake in the eight-metal solutions did not significantly deviate from those observed in the single-metal solutions. The saturation of metal removal capacity occurred within 4–5 h in the presence of all eight metals (Supplementary Figure S3). Moreover, the viability of the strains remained unaffected by the presence of metals in both single-metal and multi-metal solutions. The number of viable cells was consistent, ranging from 7.3 to 8.9 Log10 CFU g^−1^ of dry cell mass for strains R3 and R19, and from 8.0 to 9.7 Log10 CFU g^−1^ of dry cell mass for strains L2 and L30 throughout the 24-h metal binding assay (Supplementary Figure S4). There was no statistically significant difference (*p* ≤ 0.05) in the number of viable cells between the single-metal and multi-metal solutions.

### 3.3. FT-IR

**Figure 2:**
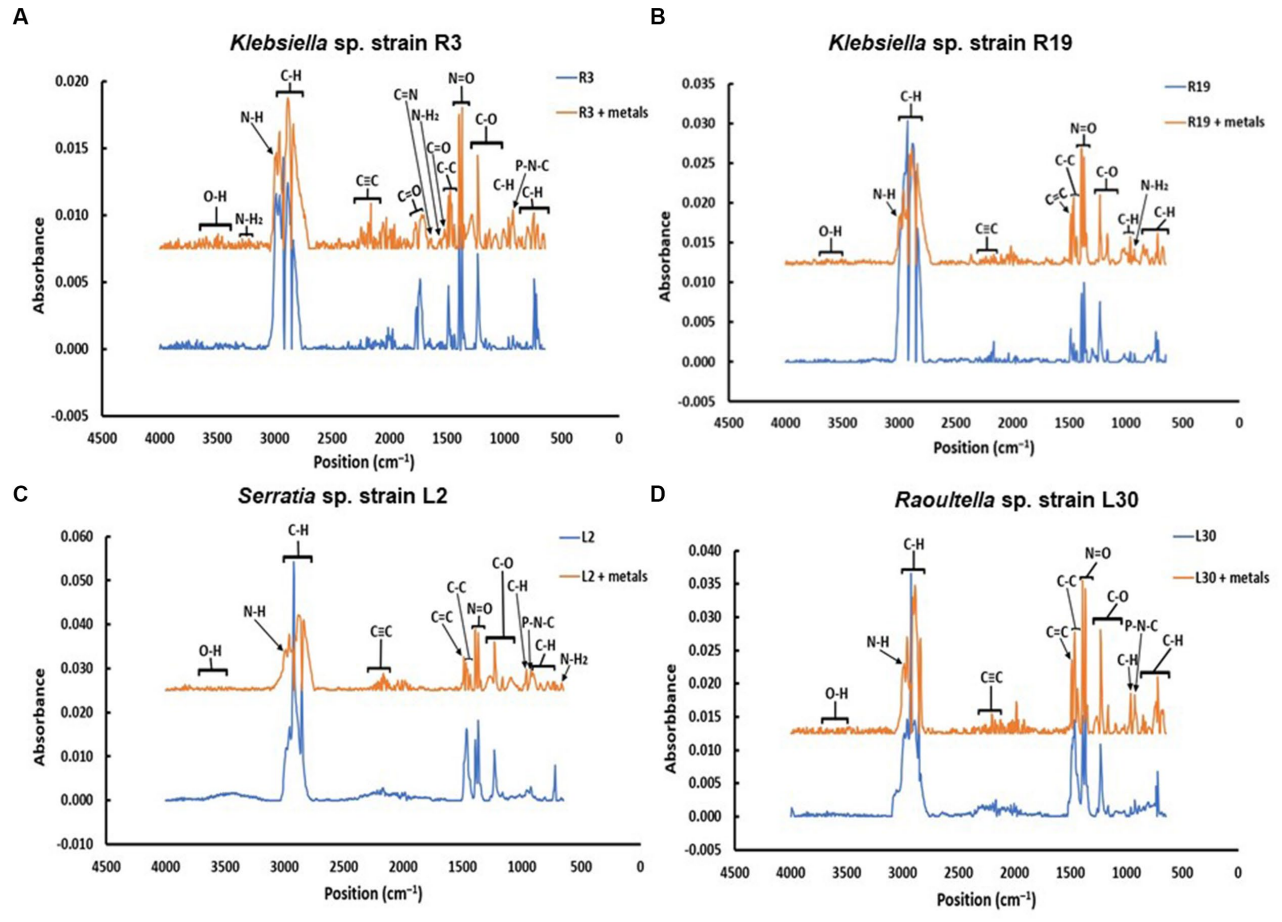
Comparison of the IR spectra of **(A)** *Klebsiella* sp. R19; **(B)** *Klebsiella* sp. R19; **(C)** *Serratia* sp. L2; and **(D)** *Raoultella* sp. L30 before and after absorption of mixed metals.

FTIR spectra were acquired for bacterial strains before and after the uptake of a combination of eight metals, spanning the range of 4,000 to 400 cm^−1^ (depicted in Figure 2). The FT-IR profiles of metal-free bacterial strains exhibited diverse peaks, revealing the intricate nature of the bacterial cell surface. Common bands were evident in the strains prior to metal uptake across all four bacterial types, albeit with fewer IR peaks observed in the *Serratia* sp. L2 and *Raoultella* sp. L30 strains (illustrated in Figures 2A,B) compared to the *Klebsiella* sp. R3 and *Klebsiella* sp. R19 strains (shown in Figures 2C,D). These IR bands corresponded to functional groups including amino (N-H, NH_2_), alkyne (C ≡ C), carbonyl (C=O), carboxylic (C-O), hydroxyl (-OH), and phosphate (P=O) groups (depicted in Figure 2). The assignments of these bands and the specific functional groups for each of the four strains are detailed in Tables 2–5. Upon exposure to metals, alterations in the intensity of bands became apparent, accompanied by shifts in absorption bands and the emergence of new peaks. The number of IR bands expanded as the strains encountered the multi-metal environment. Notably, the occurrences of IR shifts and new peaks were more pronounced in *Klebsiella* sp. R3 and *Klebsiella* sp. R19 than in *Serratia* sp. L2 and *Raoultella* sp. L30 strains. In the metal-loaded L2 and L30 strains, IR spectra shifts indicated the involvement of functional groups linked to aromatic organics, alkynes (C ≡ C), and alkanes (C-H). Conversely, the R3 and R19 strains demonstrated shifts associated with aromatic organics, alkynes (C ≡ C), alkanes (C-H), as well as hydroxyl, amine, and aldehyde functional groups. Moreover, new peaks emerged in the spectra of the R3 and R19 metal-loaded strains, indicating the presence of aromatic compounds, alkanes (C-H), carboxyl (C-C), alkynes (C ≡ C), alcohol (R-CHO), amine (P-NH, NH_2_), and hydroxyl (O-H) functional groups. These observations underscored the intricate alterations in the bacterial cell surfaces induced by the uptake of multiple metals, with distinct variations based on bacterial strain type.

**Table 2:** IR absorption band changes and possible assignment for the metal-free and metal-loaded *Klebsiella* strain sp. R3.

FTIR peak	*Klebsiella* sp. strain R3					
	Metal-free	Metal-loaded	Displacement*	Functional groups	Bond	Assignment
1		667	667	C_2_H_2_R_2_	C-H out-of-plane-bend	Alkene
2	719	712	7	1,3-Disubstituted (Aromatic compounds)	C-H out-of-plane-bends	Aromatic
3	738	742	4	1,2-Disubstituted (Aromatic compounds)	C-H out-of-plane-bend	Aromatic
4	805	798	7	C_2_HR_3_	C-H out-of-plane-bend	Alkene
5		846	846	1,4-Disubstituted (Aromatic compounds)	C-H out-of-plane-bend	Aromatic
6		876	876	1,3-Disubstituted (Aromatic compounds)	C-H out-of-plane-bend	Aromatic
7		1,003	1,003	(RCO)_2_O	C-O stretch	Carbonyl
8		1,128	1,128	R-OH	C-O stretches	Alcohol
9		1,282	1,282	RCOOR’	C-O stretch	Carbonyl
10	1,457	1,469	12	C-C	C-C bend	Alkane
11	1,558	1,557	1	P-NH_2_	NH_2_	Amine
12	1,647	1,640	7	R_2_C=NR or R_2_C=NH	C=N stretch	Imine and Oxime
13		1,700	1,700	R_2_C=O or RCOOH	C=O stretch	Ketone or carboxylic acid
14		1,718	1,718	R_2_C=O or RCOOH	C=O stretch	Ketone or carboxylic acid
15		1,770	1,770	RCOC1	C=O stretch	Acid Chloride
16	2,121	2,117	4	C ≡ C	C ≡ C stretch	Alkyne
17	2,147	2,136	11	C ≡ C	C ≡ C stretch	Alkyne
18		2,162	2,162	C ≡ C	C ≡ C stretch	Alkyne
19	2,177	2,184	7	C ≡ C	C ≡ C stretch	Alkyne
20	2,195	2,199	4	C ≡ C	C ≡ C stretch	Alkyne
21	2,251	2,259	8	RC ≡ N	C ≡ N stretch	Nitrile
22	2,859	2,861	3	C-H	C-H stretch	Alkane
23	2,986	2,984	2	P-NH	NH	Amine
24		3,120	3,120	C=C-H	C-H stretch	Alkene
25		3,224	3,224	P-NH_2_	NH_2_	Amine
26		3,280	3,280	P-NH_2_	NH_2_	Amine
27		3,493	3,493	RO-H hydrogen bond	O-H stretch	Hydroxyl
28		3,993	3,593	RO-H free	O-H stretch	Hydroxyl
29	3,630	3,623	7	RO-H free	O-H stretch	Hydroxyl

*IR band shifts in red; new bands in blue.

**Table 3:** IR absorption band changes and possible assignment for the metal-free and metal-loaded *Klebsiella* sp. strain R19.

FTIR peak	*Klebsiella* sp. strain R19					
	Metal-free	Metal-loaded	Displacement*	Functional groups	Bond	Assignment
1		675	675	C_2_H_2_R_2_	C-H out-of-plane bend	Alkene
2	716	719	3	1,3-Disubstituted (Aromatic compounds)	C-H out-of-plane bend	Aromatic
3	734	737	4	Monosubstituted	C-H out-of-plane bend	Aromatic
4		768	768	1,2-Disubstituted (Aromatic compounds)	C-H out-of-plane bend	Aromatic
5	801	813	12	C_2_HR_3_	C-H out-of-plane-bend	Alkene
6		846	846	1,4-Disubstituted (Aromatic compounds)	C-H out-of-plane-bend	Aromatic
7		924	924	P-NH_2_	NH_2_	Amine
8		939	939	P-NH_2_	NH_2_	Amine
9	962	964	2	C_2_H_2_R_2_	C-H out-of-plane-bend	Alkene
10		988	988	C_2_H_3_R	C-H out-of-plane-bends	Alkene
11		1,118	1,118	R-OH	C-O stretches	Alcohol
12	1,230	1,238	8	R-OH	C-O stretches	Alcohol
13	1,368	1,373	5	R-NO_2_	N=O	Nitro
14	1,390	1,393	3	R-NO_2_	N=O	Nitro
15	1,435	1,434	1	C-C	C-C bend	Alkane
16	1,457	1,461	4	C-C	C-C bend	Alkane
17	1,487	1,490	2	C=C	C=C	Aromatic
18		2,147	2,147	C ≡ C	C ≡ C stretch	Alkyne
19	2,166	2,162	4	C ≡ C	C ≡ C stretch	Alkyne
20	2,188	2,193	5	C ≡ C	C ≡ C stretch	Alkyne
21		2,210	2,210	C ≡ C	C ≡ C stretch	Alkyne
22		2,230	2,230	C ≡ C	C ≡ C stretch	Alkyne
23		2,232	2,232	C ≡ C	C ≡ C stretch	Alkyne
24	2,837	2,840	3	RCHO	C-H stretch	Aldehyde
25	2,881	2,889	8	C-H	C-H stretch	Alkane
26		2,911	2,911	C-H	C-H stretch	Alkane
27		2,930	2,930	C-H	C-H stretch	Alkane
28	2,926	2,960	34	C-H	C-H stretch	Alkane
29		2,986	2,986	P-NH	NH	Amine
30		2,997	2,997	P-NH	NH	Amine

*IR band shifts in red; new bands in blue.

**Table 4:** IR absorption band changes and possible assignment for the metal-free and metal-loaded *Serratia* sp. strain L2.

FTIR peak	*Serratia* sp. strain L2					
	Metal-free	Metal-loaded	Displacement*	Functional groups	Bond	Assignment
1		663	663	P-NH	NH_2_	Amine
2		704	704	Monosubstituted (aromatic compound)	C-H out-of-plane-bends	Aromatic
3	719	723	4	1,3-Disubstituted (aromatic compound)	C-H out-of-plane-bend	Aromatic
4		846	846	1,4-Disubstituted (aromatic compound)	C-H out-of-plane-bend	Aromatic
5		909	909	C_2_H_3_R	C-H out-of-plane-bends	Alkene
6		1,092	1,092	R-OH	C-O stretches	Alcohol
7		1,435	1,435	C-C	C-C bend	Alkane
8		1,469	1,469	C-C	C-C bend	Alkane
9	2,132	2,134	4	C ≡ C	C ≡ C stretch	Alkyne
10	2,154	2,158	4	C ≡ C	C ≡ C stretch	Alkyne
11	2,195	2,184	11	C ≡ C	C ≡ C stretch	Alkyne
12	2,218	2,214	4	C ≡ C	C ≡ C stretch	Alkyne
13		2,837	2,837	RCHO	C-H stretch	Aldehyde
14	2,851	2,885	34	C-H	C-H stretch	Alkane
15	2,922	2,930	8	C-H	C-H stretch	Alkane
16	2,956	2,960	4	C-H	C-H stretch	Alkane
17		2,986	2,986	P-NH	NH	Amine
18		3,496	3,496	RO-H hydrogen bond	O-H stretch	Hydroxyl
19		3,623	3,623	RO-H free	O-H stretch	Hydroxyl

*IR band shifts in red; new bands in blue.

**Table 5:** IR absorption band changes and possible assignment for the metal-free and metal-loaded *Raoultella* sp. strain L30 strain.

FTIR peak	*Raoultella* sp. strain L30					
	Metal-free	Metal-loaded	Displacement*	Functional groups	Bond	Assignment
1		678	678	C_2_H_2_R_2_	C-H out-of-plane bend	Alkene
2	734	738	4	Monosubstituted (Aromatic Compound)	C-H out-of-plane bends	Aromatic
3	805	809	4	C_2_HR_3_	C-H out-of-plane bends	Alkene
4	846	848	2	1,4-Disubstituted (Aromatic Compound)	C-H out-of-plane bends	Aromatic
5	887	886	1	C_2_H_2_R_2_	C-H out-of-plane bend	Alkene
6		1,066	1,066	(RCO)_2_O	C-O stretch	Carbonyl
7		1,260	1,260	R-OH	C-O stretches	Alcohol
8	1,461	1,465	4	C-C	C-C bend	Alkane
9		1,562	1,562	P-NH_2_	NH_2_	Amine
10		1,640	1,640	R_2_C=NR or R_2_C=NH	C=N stretch	Imine and Oxime
11		2,147	2,147	C ≡ C	C ≡ C stretch	Alkyne
12		2,162	2,162	C ≡ C	C ≡ C stretch	Alkyne
13	2,188	2,190	2	C ≡ C	C ≡ C stretch	Alkyne
14		2,210	2,210	C ≡ C	C ≡ C stretch	Alkyne
15		2,230	2,230	C ≡ C	C ≡ C stretch	Alkyne
16		2,840	2,840	RCHO	C-H stretch	Aldehyde
17	2,855	2,859	4	C-H	C-H stretch	Alkane
18	2,922	2,930	8	C-H	C-H stretch	Alkane
19		2,986	2,986	P-NH	NH	Amine
20		2,997	2,996	P-NH	NH	Amine
21		3,493	3,493	RO-H hydrogen bond	O-H stretch	Hydroxyl
22		3,586	3,586	RO-H free	O-H stretch	Hydroxyl

*IR band shifts in red; new bands in blue.

### 3.4. SEM–EDX

**Figure 3:**
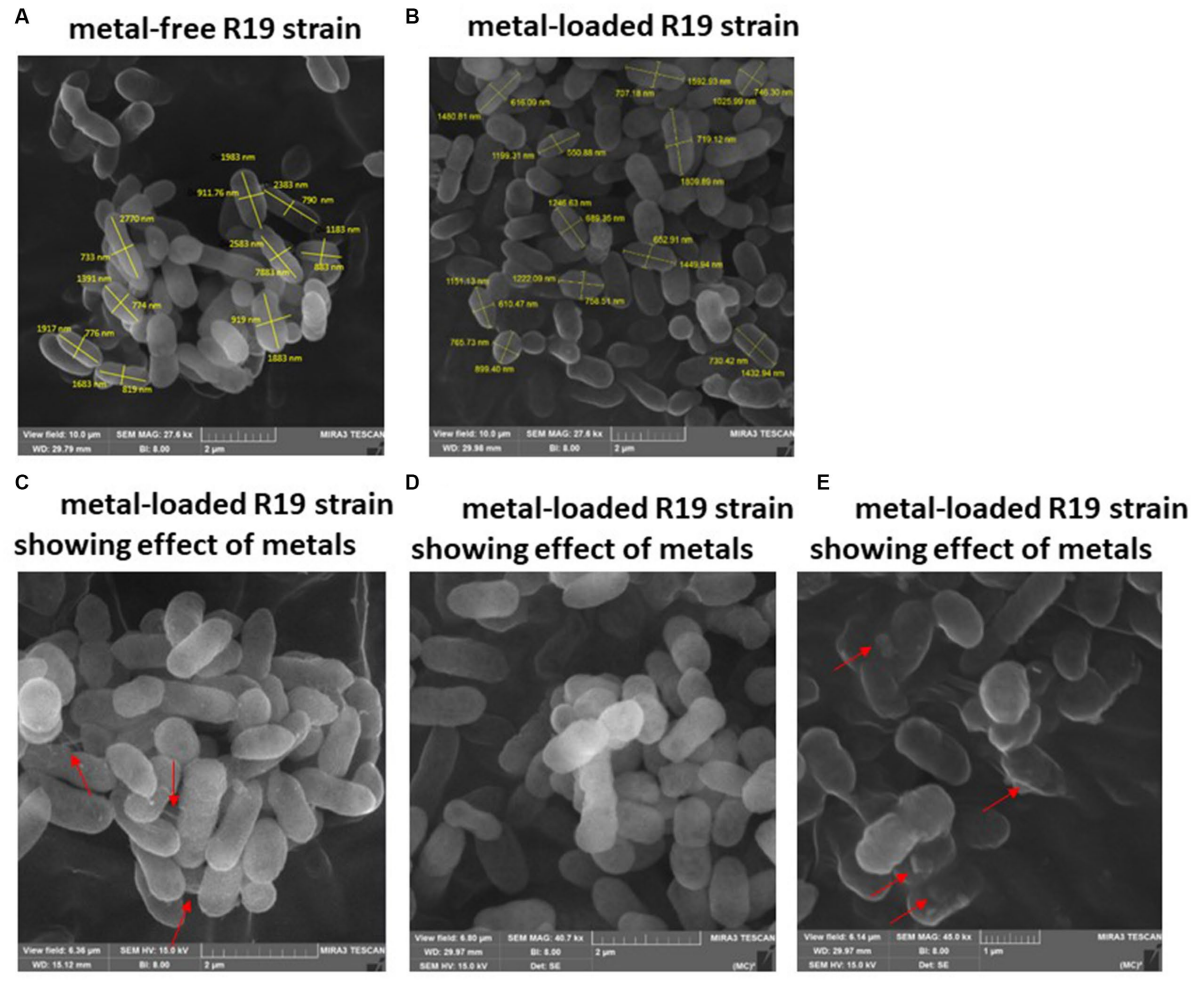
SEM images of *Klebsiella* sp. R19 before **(A)** and after absorption of mixed metals **(B)** and morphological changes when exposed to the eight metals **(C–E)**. The size (length and width) and cells are shown in nm. The red arrows point to the presence of flocs on the cell surface.

**Figure 4:**
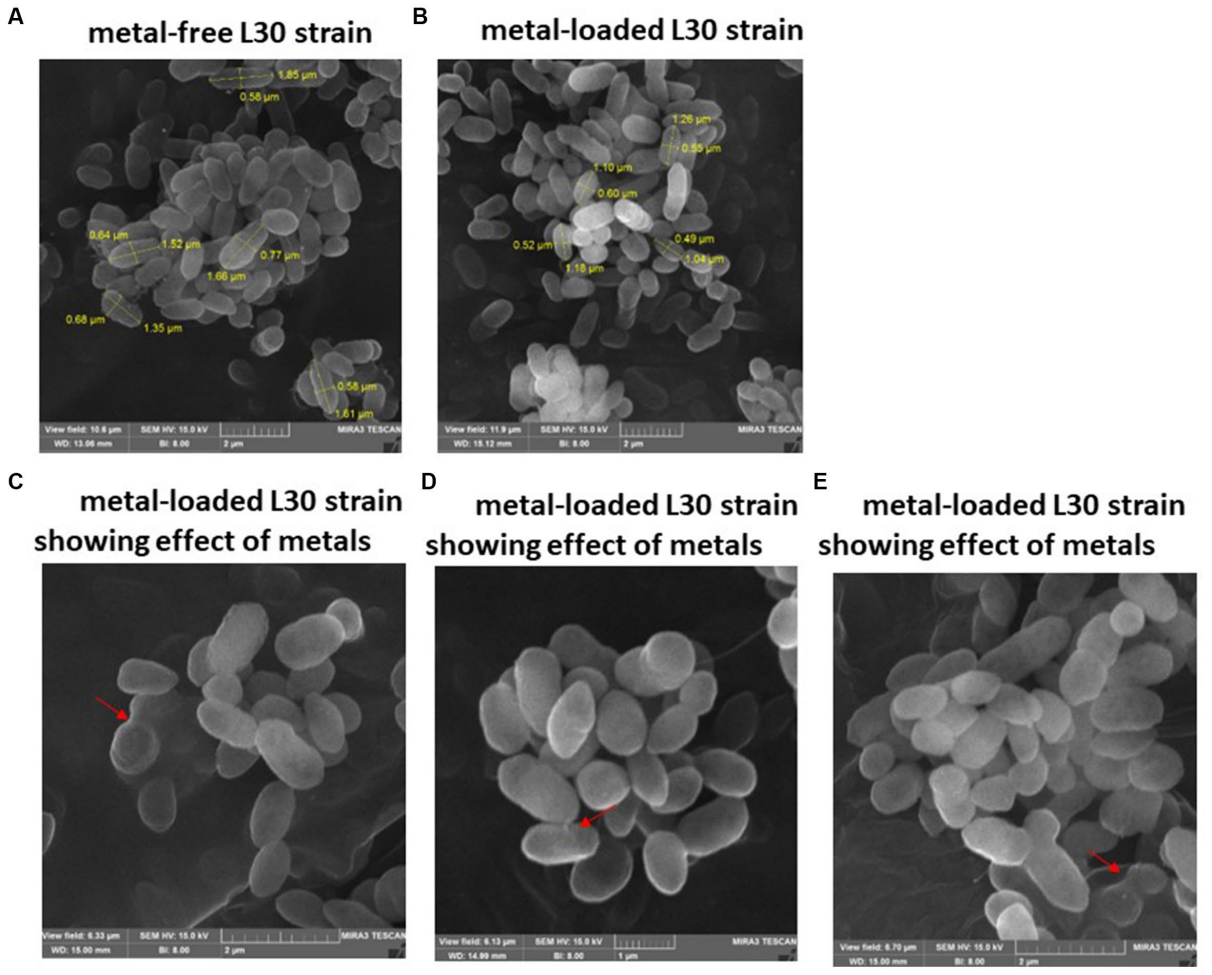
SEM images of *Raoultella* sp. L30 L30 before **(A)** and after absorption of mixed metals **(B)** and morphological changes when exposed to the eight metals **(C–E)**. The size (length and width) and cells are shown in μm. The red arrows point to the presence of flocs on the cell surface.

Examination of SEM micrographs revealed alterations in both size and morphology of *Klebsiella* sp. R19 (depicted in Figure 3) and *Raoultella* sp. L30 (depicted in Figure 4) before and after metal adsorption. Initially, *Klebsiella* sp. R19 exhibited an average length of 1.78 ± 0.40 μm and an average width of 0.71 ± 0.12 μm (Figure 3A). Subsequent to the biosorption process in an aqueous solution containing eight metals, its dimensions were diminished to 1.32 ± 0.27 μm x 0.70 ± 0.11 μm (Figure 3B), reflecting reductions of 26% in length and 3% in width. A parallel outcome was observed for *Raoultella* sp. L30, wherein its size underwent a decrease from 1.53 ± 0.34 μm x 0.65 ± 0.09 μm (Figure 4A) to 1.17 ± 0.27 μm x 0.70 ± 0.07 μm (Figure 4B). SEM analysis also provided insights into notable modifications observed on the cell surfaces of both *Klebsiella* sp. R19 and *Raoultella* sp. L30 bacteria Following the process of metal biosorption. The cell surfaces of *Klebsiella* sp. R19 exhibited a distinctive texture characterized by irregularities, including dents, wrinkles, and elongations (Figures 3C,D). These surface features appeared to be a direct consequence of the interaction between the bacterial cells and the adsorbed metals. The metals’ toxic influence appeared to induce these alterations in the cell surface structure. Additionally, a considerable amount of flocculation was evident on the cell surfaces. This phenomenon could be attributed to the secretion of extracellular polysaccharides prompted by the presence of metals. The extracellular polysaccharides might have contributed to the aggregation of bacterial cells, leading to the formation of these flocculent clusters (Figure 3C). The cell surfaces of *Raoultella* sp. exhibited varying degrees of deformations, creases, or elongations due to the adverse impact of the metals. Additionally, a notable amount of flocculation emerged on the cell surfaces, prompting cellular aggregation (Figures 4C,D). This phenomenon might be attributed to the metal-induced secretion of extracellular polysaccharides (Gupta and Diwan, 2017).

**Figure 5:**
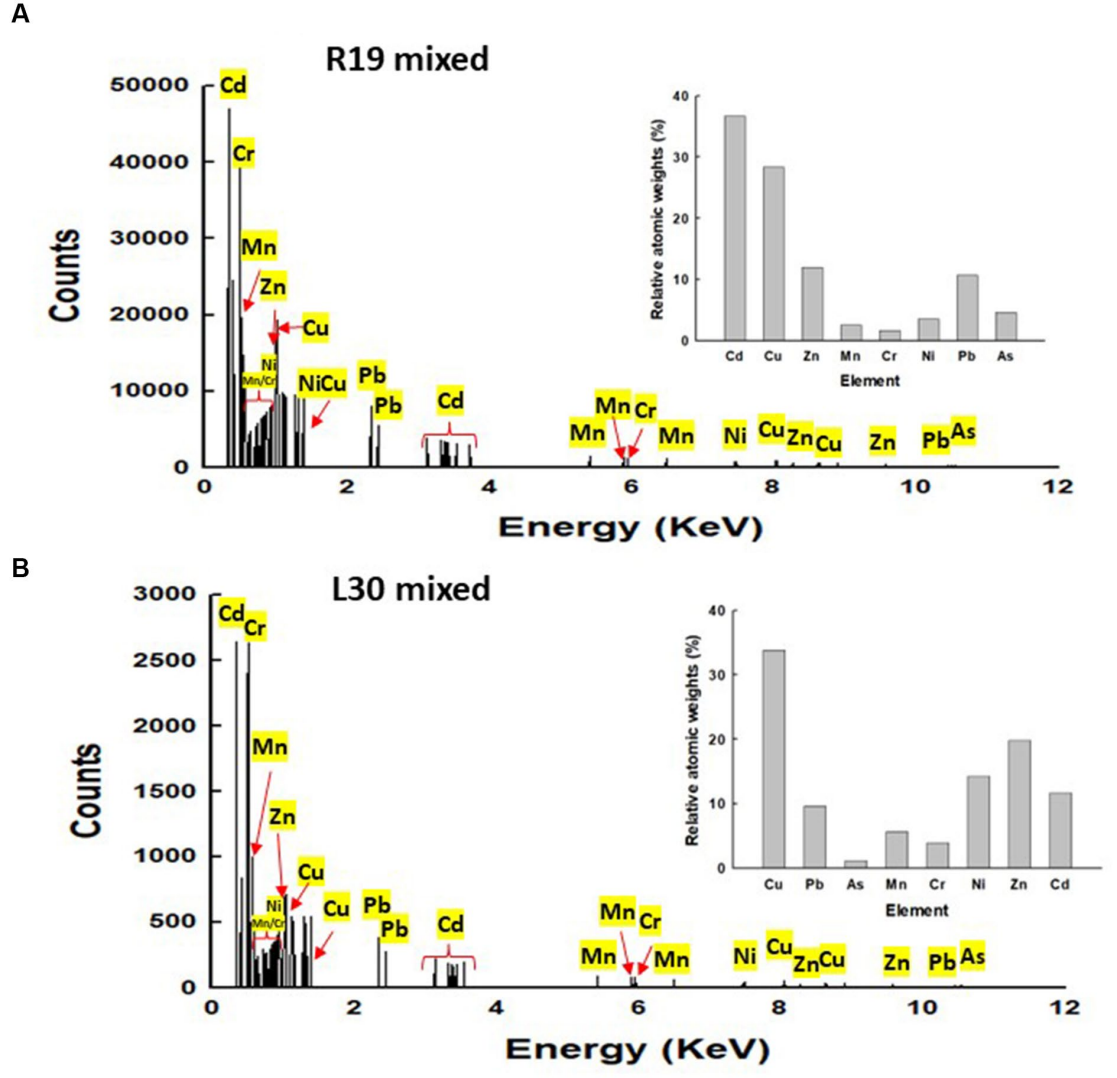
Energy dispersive X-ray spectroscopy (EDX) results for **(A)** *Klebsiella* sp. R19 and *Serratia* sp. L30 after absorption of mixed metals **(B)**. Quantification results of Pb, As, Cd, Cr, Cu, Mn, Ni or Zn in *Klebsiella* sp. R19 and *Serratia* sp. L30 strains at 15 kV.

The energy-dispersive X-ray spectroscopy (EDX) analysis of *Klebsiella* sp. R19 and *Serratia* sp. L30 (Figure 5) affirmed the presence of adsorbed metals on the cell surfaces. The quantified outcomes, illustrating the distribution of the eight metals on the cell surfaces of *Klebsiella* sp. R19 and *Raoultella* sp. L30, are presented in Figure 5. Among these eight metals, Cd^2+^ predominated the cell surface of *Klebsiella* sp. followed by Cu^2+^, Zn^2+^ and Pb^2+^ (Figure 5A), while Cu^2+^ stood out as the foremost element, followed by Zn^2+^, Cd^2+^, and As^3+^ (Figure 5B).

### 3.5. STEM-EDX

**Figure 6:**
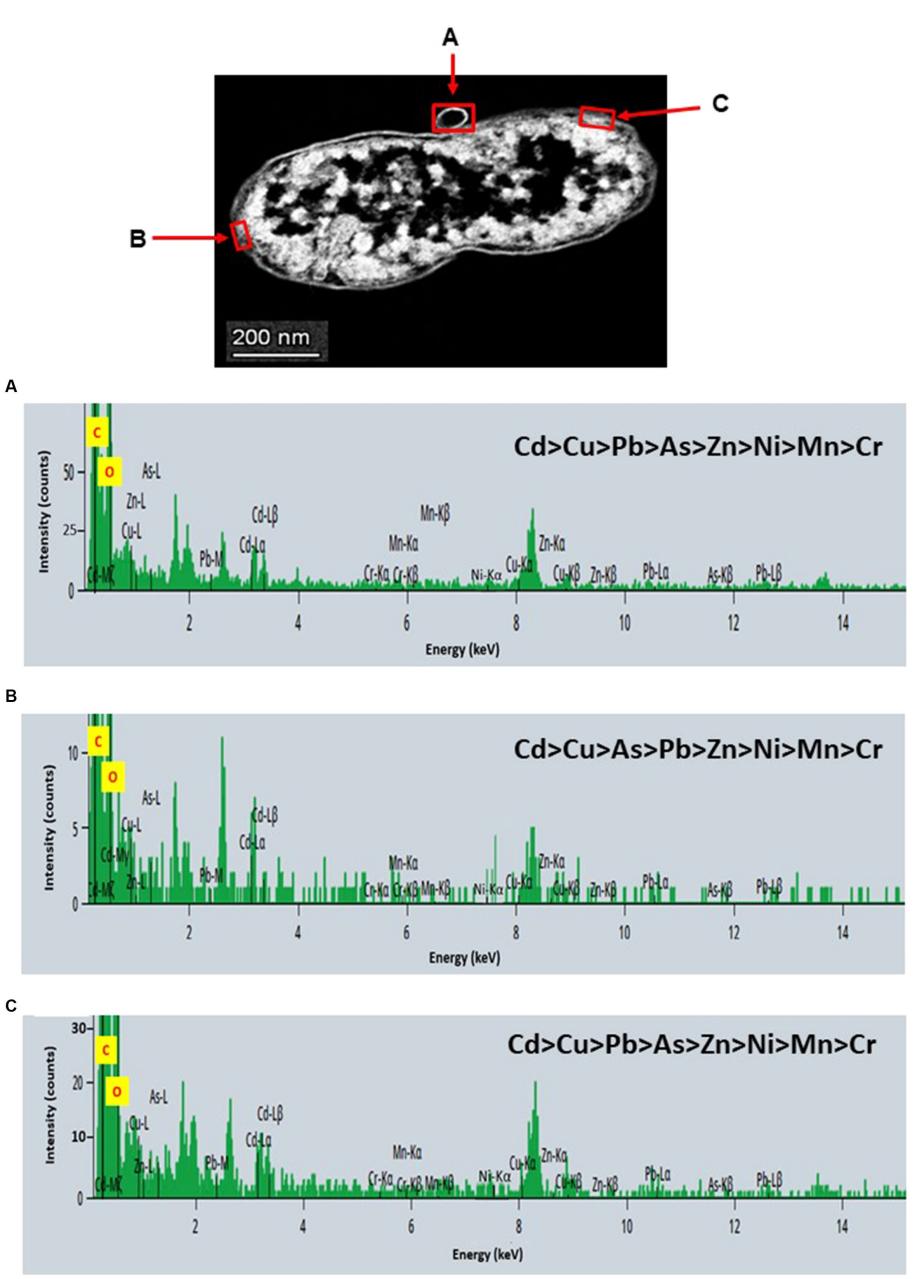
Scanning transmission electron micrographs (STEM) off a thin section of metal-loaded *Klebsiella* sp. R19 and the location of metals. **(A–C)** Energy dispersive X-ray spectra of the surface biosorption of metals acquired from the region indicated by arrow A, B, and C. The electron-dense granules appear bright on the STEM-HAADF image.

**Figure 7:**
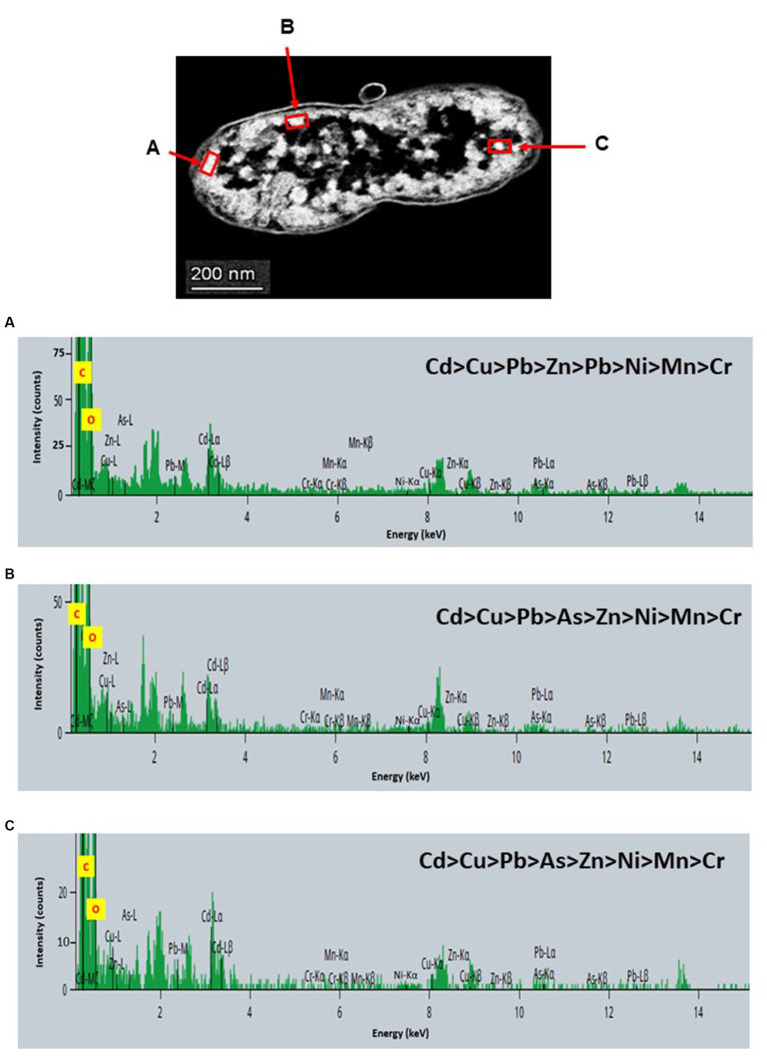
Scanning transmission electron micrographs (STEM) off a thin section of metal-loaded *Klebsiella* sp. R19 and the location of metals. **(A–C)** Energy dispersive X-ray spectra of the intracellular accumulation of metals acquired from the region indicated by arrow A, B, and C. The electron-dense granules appear bright on the STEM-HAADF image. Granules are of three types: large and compact (blue arrows), small (green arrows), and saturated around storage granules (purple arrow).

**Figure 8:**
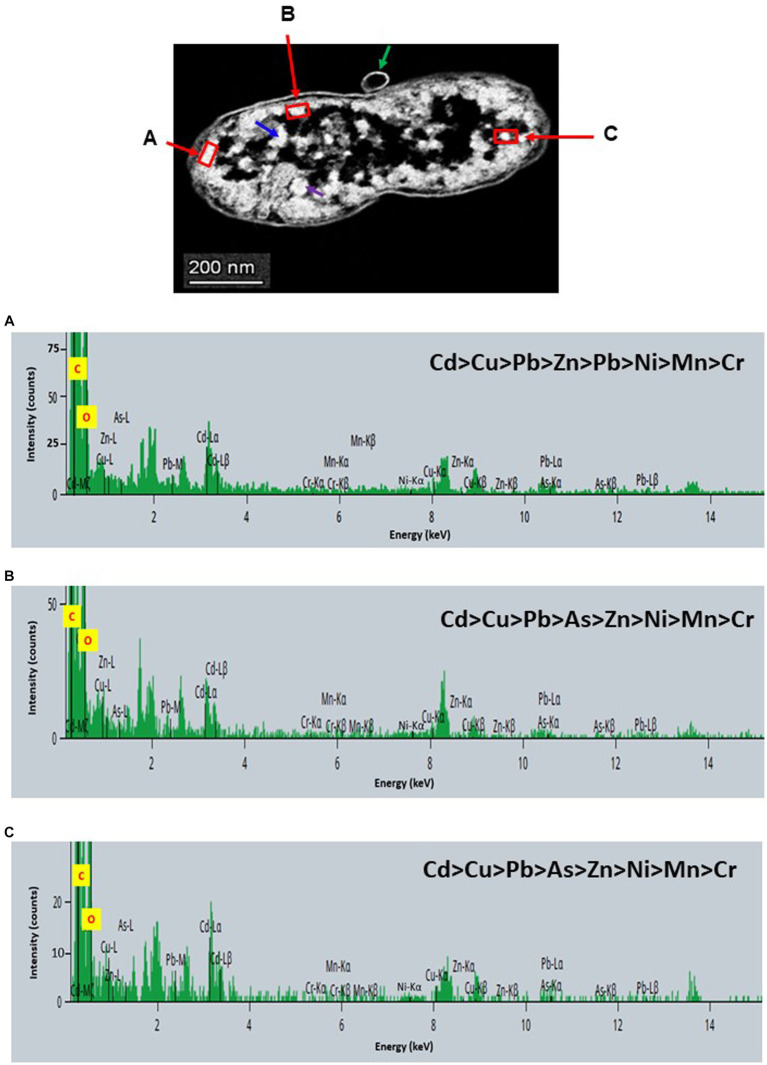
Scanning transmission electron micrographs (STEM) off a thin section of metal-loaded *Raoultella* sp. L30 and the location of metals. **(A–C)** Energy dispersive X-ray spectra of the surface biosorption of metals acquired from the region indicated by arrow A, B, and C. The electron-dense granules appear bright on the STEM-HAADF image.

**Figure 9:**
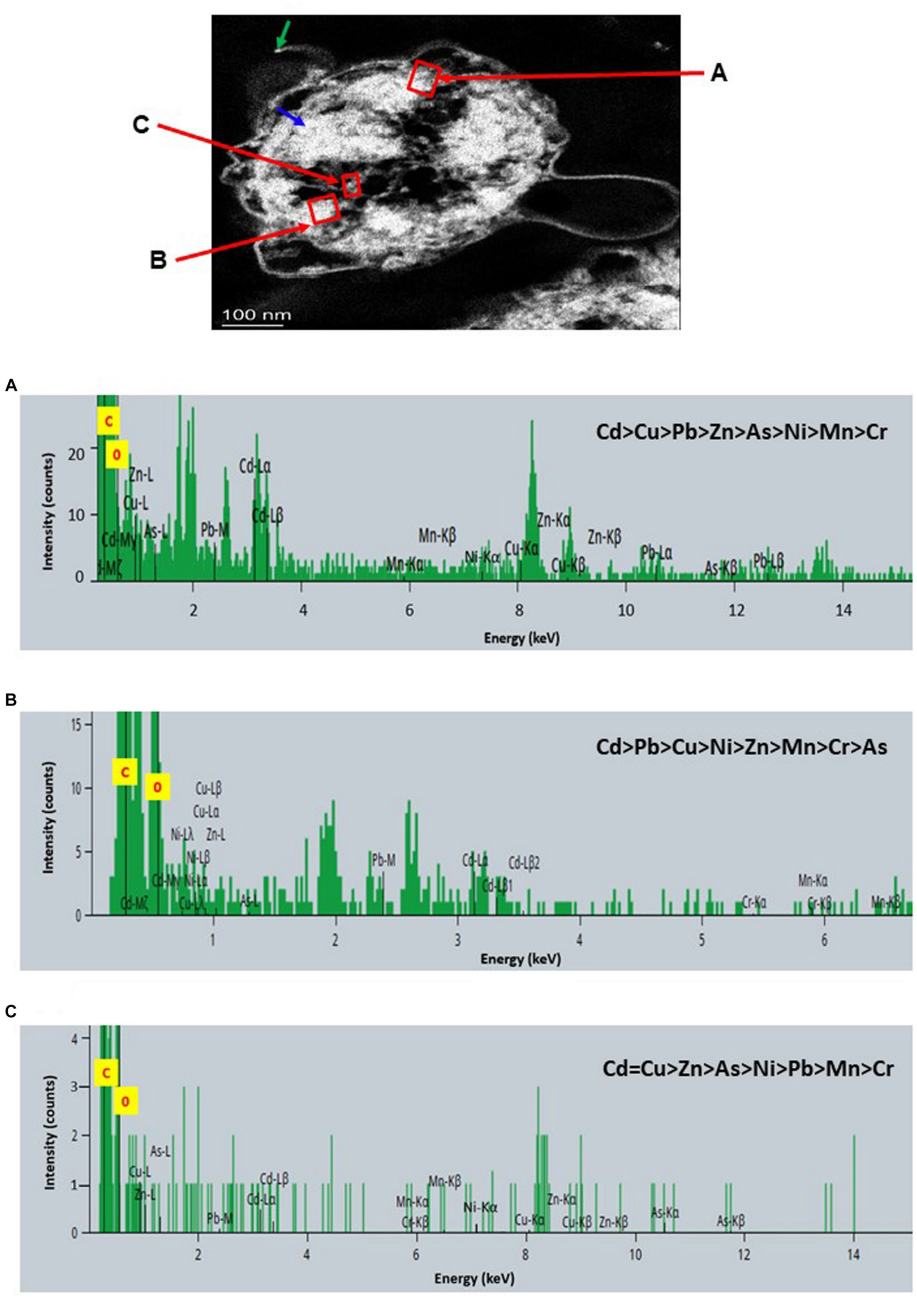
Scanning transmission electron micrographs (STEM) off a thin section of metal-loaded *Raoultella* sp. L30 and the location of metals. **(A–C)** Energy dispersive X-ray spectra of the intracellular accumulation of metals acquired from the region indicated by arrow A, B, and C. The electron-dense granules appear bright on the STEM-HAADF image. Granules are of three types: large and compact (blue arrows), small (green arrows), and saturated around storage granules (purple arrow).

STEM images unveiled the accumulation of metals within both the cytoplasm and cell walls of *Klebsiella* sp. R19 (depicted in Figures 6, 7) and *Raoultella* sp. L30 (illustrated in Figures 8, 9). Numerous electron-dense granules were discernible, situated across cell walls, membrane fractions, and within the cytoplasm. These electron-dense granules appeared vividly in the STEM-HAADF image. The granules exhibited three distinct types: sizable and compact granules (indicated by blue arrows), smaller granules (marked by green arrows), and saturation clustering around storage granules (designated by purple arrow) (depicted in Figure 7). Interestingly, *Raoultella* sp. L30 did not exhibit evident storage granules. The verification via EDX analysis affirmed the presence of metal accumulation on the cell wall, cell membrane, and cytoplasm. The position beam spectra clearly indicated the detection of all eight metals within the electron-dense granules for both strains (portrayed in Figures 6–9). These electron-dense granules encapsulate metal complexes, effectively binding the metals. To determine the relative distribution of the eight metals within the electron-dense granules, the atomic percentages (atm %) of As^3+^, Pb^2+^, Cu^2+^, Mn^2+^, Zn^2+^, Cd^2+^, Cr^6+^, and Ni^2+^ were normalized to 100%, after excluding contaminants (such as C, O, etc.).

For *Klebsiella* sp. R19, Cd overwhelmingly dominated the electron-dense granules within the cell wall and cell membrane. The order of metal distribution was as follows: Cd^2+^ > Cu^2+^ > Pb^2+^ > As^3+^ > Zn^2+^ > Ni^2+^ > Mn^2+^ > Cr^6+^ (depicted in Figure 6). A similar pattern emerged for the electron-dense granules within the cytoplasm (illustrated in Figure 4). Meanwhile, in the case of *Raoultella* sp. L30, the electron-dense granules in the cell wall [Cu^2+^ > Cd^2+^ > Pb^2+^ = Zn^2+^ > Cr^6+^ > Ni^2+^ > Mn^2+^ > As^3+^] and cell membrane [Cu^2+^ > Pb^2+^ > Cd^2+^ > Zn^2+^ > Cr^6+^ > Ni^2+^ > Mn^2+^ > As^3+^] were notably enriched in Cu^2+^ (depicted in Figure 8). While the relative metal distribution in the electron-dense granules within the cytoplasm of *Raoultella* sp. L30 exhibited some variability (shown in Figure 9), the dominant metal consistently observed in these granules was Cu^2+^ (as shown in Figure 9).

## 4. Discussion

Metal tolerance in bacteria is an essential trait and may help in developing an effective process for the treatment of heavy metal contaminated wastewaters. The results of the MIC assay provided insights into the range of metal concentrations at which these strains could withstand metal exposure. The strains were adapted to high concentrations of not only Pb but also As^3+^, Mn^2+^, and Zn^2+^ (Table 1). From a broader perspective, it is notable that the bacterial strains exhibited considerable resistance against certain metals. Specifically, they demonstrated robust resilience against As^3+^, with MICs ranging from 250 to 450 mg L^−1^. Similarly, they showcased noteworthy tolerance against Pb^2+^, spanning from 700 to 800 g L^−1^. Moreover, the strains exhibited impressive resilience against Mn concentrations exceeding 2,000 mg L^−1^, as well as Zn^2+^ concentrations ranging from 500 to 1,100 mg L^−1^. These findings suggest that these bacterial strains have evolved mechanisms to withstand and adapt to these specific heavy metals. Conversely, the strains exhibited comparatively lower tolerance toward other heavy metals. Cd^2+^, Cr^6+^, Cu^2+^, and Ni^2+^ elicited relatively lower MIC values, indicating that the bacterial strains are more sensitive to these metals. This variability in sensitivity across different metals underscores the complexity of bacterial responses to heavy metal exposure. it becomes evident that strains L2 and L30 (*Serratia* sp. strain L2 and *Raoultella* sp. strain L30) displayed heightened levels of metal tolerance compared to strains R3 (*Klebsiella* sp.) and R19 (*Klebsiella* sp.). This disparity in metal tolerance could stem from a multitude of factors, such as variations in the strains’ genetic makeup, metabolic pathways, and the presence of specific metal detoxification mechanisms (Rosen, 1995; Silver, 1996; Nies, 1999; Rensing and Grass, 2003; Silver and Phung, 2005; Helbig and Grass, 2017).

The biosorption abilities of bacterial isolates in both single metal and multi-metal solutions, shedding light on their potential effectiveness in metal removal and the intricate dynamics of simultaneous metal exposure. In the context of single metal solutions, Within the realm of single metal solutions, the capabilities of four metal-tolerant strains emerge prominently. These strains demonstrated impressive efficacy in extracting substantial concentrations of diverse metal ions, including Pb^2+^, As^2+^, Zn^2+^, Cd^2+^, Cr^6+^, and Mn^2+^—each exceeding 50 mg of metal per gram of cell dry mass. Notably, strains R3 and R19 exhibited heightened metal cation removal compared to L2 and L30. These findings underscore the strains’ adaptability and potential applicability in targeted scenarios where specific metal pollutants predominate. Transitioning into solutions encompassing eight metals, a more intricate picture unfolds. The co-presence of multiple metal cations elicited inhibitory effects in the cation binding process, stemming from the competition for binding sites on both bacterial cell surfaces and extracellular polymeric substances (EPS). It has been reported that in solutions containing multiple metals, some of the metal cations already bound on the EPS exert a strong hindrance to the access of other cations at the adjacent adsorption sites (Sag et al., 2000; Micheletti et al., 2008; Pradhan and Rai, 2011; Lu et al., 2021). This steric effect results in EPS conformational change (Huang et al., 2022) that leads to the general reduction in metal removal. *Klebsiella* sp. R19 showed the highest affinity to Pb^2+^ in mixed metal solutions with *q* value even higher than in single-metal solutions (Supplementary Figure S2). In contrast to Pb^2+^ the other seven metals performed poorer in the multiple-metal tests. The reduction in metal removal could be because the presence of many metals may have overwhelmed the cells and EPS to a point that they cannot bind to as many metal cations as in the single-metal tests. Nonetheless, *Klebsiella* sp. R19 was the strain that removed the most metal cations of all the four bacterial strains tested. In the mixed metal solutions, *Klebsiella* sp. R3, *Klebsiella* sp. R19 showed higher metal cation removal efficiencies than *Serratia* sp. L2 and *Raoultella* sp. L30. Insights from the study align with Bowman et al.’s (2018) observations, linking higher flocculation activities of bacterial strains to enhanced metal cation removal capacity. Both *Klebsiella* sp. R3 and *Klebsiella* sp. R19 exhibited superior flocculation activities (79 and 71%, respectively) compared to *Serratia* sp. L2 and *Raoultella* sp. L30 (L2 = 56%; L30 = 67%). This correlation underscores that flocculation activity directly influences the EPS’s ability to bind metal ions, thus strengthening the strains’ metal removal potential.

**Figure 10:**
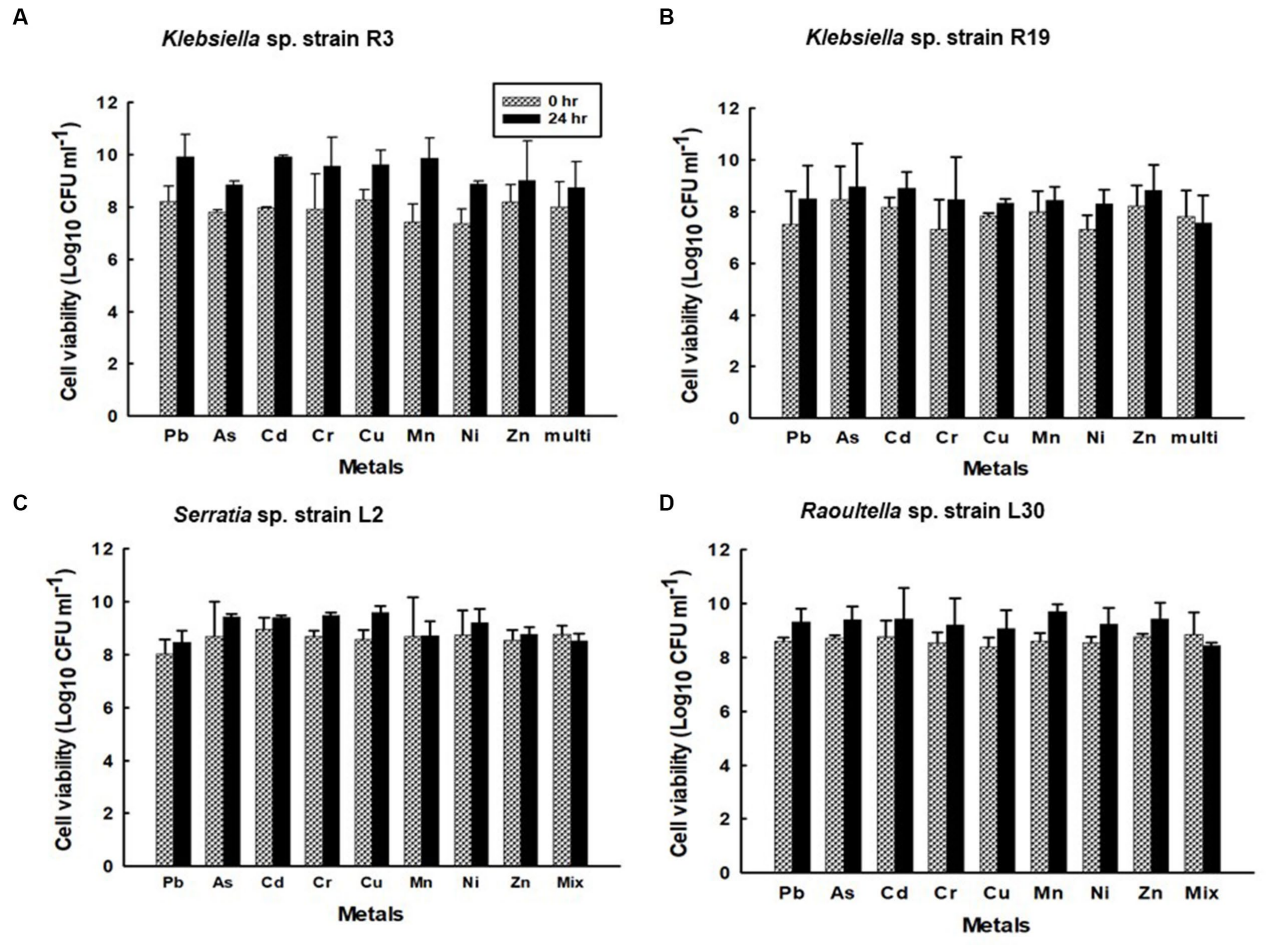
Viability of the confined **(A)** *Klebsiella* sp. R3; **(B)** *Klebsiella* sp. R19; **(C)** *Serratia* sp. L2; and **(D)** *Raoultella* sp. L30 strains during sorption in aqueous solutions single metal solutions containing Pb, Cu, Cd, Ni, As, Zn, Cr, Mn, or in mixed metal solutions containing eight metals.

Surprisingly, despite R3 and R19 strains showcasing lower metal tolerance than L2 and L30 strains (Table 1), they exhibit a remarkable ability to efficiently remove a greater quantity of metals (Figure 10). Overall, *Klebsiella* sp. R3, *Klebsiella* sp. R19 had higher metal cation removal efficiencies than *Serratia* sp. L2 and *Raoultella* sp. L30 in single- and multi-metal solutions. These findings challenge the conventional belief that metal tolerance dictates the efficacy of metal removal by bacterial strains. The results underline that metal removal efficiency is a complex phenomenon, shaped not only by a strain’s ability to endure metal stress but also by its capacity to effectively bind metal cations. The high flocculant-production ability of R3 and R19 strains (Bowman et al., 2018) emerges as a crucial factor, enabling them to interact with a higher number of metal cations in aqueous solutions. Bowman et al. (2018) suggest that the efficiency of metal removal is linked to the strains’ capability to create contact with metal cations, a vital aspect often overshadowed by metal tolerance considerations. A pertinent facet of metal resistance is its association with decreased metal uptake or impermeability, as discussed by Gadd (1990). The lower overall metal uptake observed in the L2 and L30 strains may be attributed to their metal resistance mechanisms, which inadvertently diminish their metal uptake capacity. Conversely, R3 and R19 strains’ ability to effectively remove metals could be attributed to their dynamic interaction with metal cations, potentially due to a different balance between metal resistance and efficient metal removal strategies. R3 and R19 strains might possess a higher number or more accessible functional groups, recognized as crucial metal binding sites.

To understand the interactions between bacterial cell surfaces and multiple metal ions, the four strains were subjected to a comprehensive FTIR analysis, both before and after exposure to a complex blend of eight metal ions. When metals first interact with the cell surface, they get sequestered on the surface via adsorption. This could be a result of surface complexation of the metals with the functional groups present on the cell wall (Wang et al., 2021). As a result, changes in the absorption peaks of the spectrum were witnessed in this study (Tables 2–5). The bacterial strains devoid of metal ions revealed a nuanced spectrum, marked by a multitude of absorption peaks. This intricate profile reflects the complex composition of the cellular biomass. A robust and expansive FTIR region, spanning from 3,600 to 2,500 cm^−1^, is emblematic of the vibrational stretches encompassing C-H (alkane), OH (hydroxyl), and N-H (amine) stretches (Panda et al., 2007). The distinctive C ≡ C stretch between 2,260 and 2,100 cm^−1^ aligns with alkynes, while the span from, while the span from 1,500 to 900 cm^−1^ encapsulates the overlap of C-C, C-O, and C-O-P stretches, characteristic of cellular polysaccharides (Wolkers et al., 2004). Notably, bands resonating within the range of 860 to 700 cm^−1^ correspond to aromatic organic components, further mirroring the intricate nature of cellular constitution (Grube et al., 2008). It is important to note that the appearance of a new peak at 3,496 cm^−1^ for *Serratia* sp. L2 and 3,493 for *Raoultella* sp. L30 appeared after contact with metals and these shifts may be due to Cd^+2^, Cr^2+^, Cu^2+^, or Ni^2+^ cationic interaction with the hydroxyl group for metal oxygen binding (Panda et al., 2007). The appearance of new peaks at 3,224 and 3,280 cm^−1^ for *Klebsiella* sp. R3, and 1,118, 2,986, and 2,997 cm^−1^ for *Klebsiella* sp. R19 can be attributed to the presence of phosphate and amino groups and are linked to the presence of phosphate and amino groups, playing a pivotal role in the biosorption of metals (Sodhi et al., 2020).

The appearance of new IR peaks within the spectra can be attributed to the dynamic response of bacterial strains to metal exposure, resulting in the production of extracellular polymeric substances (EPS). This phenomenon has garnered the attention of numerous researchers who have noted enhanced EPS production in bacterial cells under metal-amended conditions (Redmile-Gordon and Chen, 2017; Rizvi et al., 2019a). This observation underscores the notion that metal-induced stress triggers heightened EPS production, potentially acting as a protective layer against the deleterious effects of metal toxicity (Mathivanan et al., 2021a). EPS are such complex blends of high molecular weight polyanionic polymers, such as proteins, humic acids, polysaccharides, and nucleic acids that bind cationic metals with different degrees of specificity and affinity (Bhaskar and Bhosle, 2006; Pal and Paul, 2008; Tiquia-Arashiro, 2018; Pagliaccia et al., 2022). The multifaceted functions of EPS span beyond metal binding, extending to the safeguarding of cells against environmental stressors and the augmentation of metal biosorption capabilities (Planchon et al., 2013; Rajaram et al., 2013; Deepika et al., 2016). Notably, Mathivanan et al. (2021b) conducted FT-IR analysis on EPS produced by *Bacillus cereus* KMS3-1, revealing the presence of hydroxyl, carboxyl, or carbonyl groups and glycosidic bonds—a testament to the diversity of functional groups contributing to metal binding. Insightful research by Shuhong et al. (2014) shed light on the metal-binding mechanism, emphasizing that this process occurs on the EPS surface. Their findings indicated the pivotal roles of functional groups such as O–H, CH, C=O, C–O, and C–C=O in the binding of metal ions like Cd^+2^, Cu^2+^, and Pb^2^. Notably, these very functional groups were evident in the metal-loaded bacterial cells in the current study, as exemplified in Tables 2–5. The distinctive spectrum alterations and emergence of new bands, particularly prominent in *Klebsiella* sp. R3 and *Klebsiella* sp. R19 (Tables 2, 3), allude to the abundance of binding sites on the cell surface for metal interactions. Consequently, this abundance of interaction sites aligns with the higher observed metal removal capacity in these strains compared to *Serratia* sp. L2 and *Raoultella* sp. L30 (Figure 1). In essence, the appearance of novel IR peaks underscores the dynamic interplay between bacterial strains and metal ions, triggering EPS production and instigating a repertoire of metal-binding functional groups. This mechanism not only shields cells against metal toxicity but also enhances their metal removal prowess, accentuating the multifaceted nature of microbial-metal interactions.

The investigation into the effects of metal exposure on bacterial strains was enriched by employing SEM and EDX analyses. These techniques provided valuable insights into the morphological changes and metal adsorption patterns exhibited by the bacterial strains *Klebsiella* sp. R19 and *Raoultella* sp. L30, both before and after exposure to a complex mixture of eight metal ions. The SEM micrographs unveiled significant alterations in the size and morphology of the bacterial strains following metal adsorption. The initial dimensions of *Klebsiella* sp. R19 and *Raoultella* sp. L30 were reduced after the biosorption process, indicating a potential interaction between the bacterial cells and the adsorbed metals. These changes in size could be attributed to the toxic effects of metals on the cells, leading to structural modifications on the cell surface (Rizvi et al., 2019b). Particularly noteworthy were the distinct textures observed on the cell surfaces of both strains. The irregularities such as dents, wrinkles, and elongations reflected the complex interplay between bacterial cells and metal ions. The appearance of these surface features was likely driven by the binding of metals to specific functional groups on the bacterial cell surfaces. Dadrasnia et al. (2015) noted that the roughness and wrinkling of the cells could also be due to sequestration and precipitation of functional groups on the cell wall. The EDX analysis confirmed the presence of metals on the surface of the cells. The observed phenomenon of flocculation on the cell surfaces of both strains further underscored the intricate response to metal exposure. This flocculation could be linked to the secretion of extracellular polysaccharides as a protective mechanism against metal toxicity. The aggregation of bacterial cells resulting from this flocculation indicated a dynamic adaptation to the metal stress environment. The SEM analysis thus provided a visual representation of the cellular changes induced by metal exposure, shedding light on the intricate dynamics of bacterial response to multi-metal conditions. Moreover, EDX analysis validated the presence of adsorbed metals on the cell surfaces. The quantification of metal distribution further illuminated the preference of certain metals for binding to the cell surfaces. The dominance of specific elements on the cell surface, such as Cd^2+^ and Cu^2+^, highlighted the varying degrees of metal binding affinity exhibited by the strains. These findings corroborated the importance of examining the interaction between bacterial cell surfaces and metal ions in multi-metal environments.

The comprehensive understanding of metal uptake and accumulation within bacterial cells has been facilitated by STEM EDX analysis. This advanced analytical approach not only confirmed the presence of metals on the cell surface but also provided valuable insights into the intracellular accumulation of these metals. The coexistence of metal ions on both the cell wall and within the cytoplasm suggests intricate interactions and potential bioaccumulation mechanisms at play. The identification of metal accumulation within the cytoplasm holds significance as it hints at the presence of internalized mechanisms for metal uptake. This phenomenon has been reported in various microorganisms and underpins the complexity of microbial-metal interactions. This is particularly evident from studies conducted by different researchers (Perdrial et al., 2008; Ozdemir et al., 2012; Diba et al., 2021; Li et al., 2021), indicating the universality of intracellular metal accumulation mechanisms. However, the process of intracellular absorption is considered slower and more intricate compared to the relatively straightforward cell surface absorption (Ma et al., 2022). The initial binding of metal ions to the cell surface is often a precursor to their subsequent transport into the cell. Many established mechanisms of metal transport hinge upon the electrochemical proton gradient existing across the cell membrane. This gradient, comprising a chemical component in the form of the pH gradient and a membrane potential, actively facilitates the transport of ionized solutes across cellular membranes. This process has been explored in prior research (Borst-Pauwels, 1981; Ma et al., 2022), underscoring the critical role of membrane electrochemistry in mediating metal movement within cells. It’s noteworthy that intracellular metal uptake may also transpire via diffusion, especially in scenarios where the toxic influence of the metal leads to alterations in membrane permeability. These adaptive changes in cellular structure may inadvertently facilitate metal penetration, a phenomenon explored in studies by Gadd (1988). This underscores the multifaceted nature of microbial responses to metal exposure, wherein cells dynamically adjust their mechanisms to counteract toxicity. Comparing the metal distribution patterns between *Klebsiella* sp. R19 and *Raoultella* sp. L30 reveals intriguing differences. While both species exhibit a preference for Cu^2+^ accumulation, *Klebsiella* sp. R19 displays a unique preference for Cd^2+^, whereas *Raoultella* sp. L30 shows a more varied distribution in the cytoplasmic granules. These patterns might reflect the adaptation of each species to its specific environmental conditions, suggesting that metal availability and toxicity play crucial roles in shaping their metal accumulation strategies. The observed metal distribution patterns could be attributed to the presence of specialized metal transporters or binding proteins within these bacteria. The differences in distribution might also arise from variations in the metal-binding affinities of their cellular components.

## 5. Conclusion

The bacterial strains in the study effectively removed various metal cations, both in single- and multi-metal solutions. They exhibited both heavy metal-removing capabilities and the ability to thrive over a wide range of metal concentrations, making them suitable for potential use in metal remediation in bioreactors or *in situ* applications. Strains R3 and R19, despite being less metal-tolerant than L2 and L30 strains, demonstrated efficient metal removal because possess a higher number and more accessible carboxyl and amide functional groups, which are crucial for metal binding. This study confirmed that the simultaneous presence of multiple metals in an aqueous solution can lead to mutual inhibition in metal adsorption by the extracellular polymeric substance (EPS), resulting in reduced overall metal uptake. FTIR and SEM–EDX techniques confirmed the interactions between metal ions and functional groups on the surface of the strains. Moreover, TEM-EDX analysis showed the presence of metals on the cell surface and the cytoplasm.

Collectively, these findings advance our understanding of the metal removal capabilities and adaptive mechanisms of bacterial strains. They underscore the potential of these strains for applications in environmental remediation and the treatment of metal-contaminated effluents. Furthermore, the study emphasizes the critical role of tailoring strain selection to suit the specific metal composition of effluent environments, thus enhancing the efficiency and effectiveness of metal removal strategies.

## 6. Future studies

Future research should explore the practical applications of the highly efficient metal-resistant bacterial strains identified in this study by conducting pilot-scale studies and field trials to assess their real-world performance in diverse contaminated environments. Additionally, efforts should be directed toward optimizing bioremediation techniques and strategies that harness these strains, including the design of specialized bioreactor systems and the evaluation of scalability. Finally, investigating genetic and metabolic engineering approaches to enhance the metal-binding capabilities of these strains could lead to the development of even more specialized and efficient bioremediation tools.

## Data availability statement

The datasets presented in this study can be found in online repositories. The names of the repository/repositories and accession number(s) can be found in the article/Supplementary material.

## Author contributions

GP: Data curation, Formal analysis, Investigation, Software, Validation, Writing – original draft. DO: Data curation, Formal analysis, Investigation, Methodology, Writing – review & editing. YC: Data curation, Formal analysis, Investigation, Methodology, Validation, Writing – review & editing. AF: Formal analysis, Investigation, Methodology, Validation, Writing – review & editing. FS: Data curation, Formal analysis, Investigation, Methodology, Validation, Writing – review & editing. JF: Data curation, Formal analysis, Investigation, Methodology, Software, Writing – review & editing. RS: Data curation, Formal analysis, Methodology, Writing – review & editing. CS: Data curation, Formal analysis, Investigation, Methodology, Software, Validation, Writing – review & editing. AK: Data curation, Formal analysis, Investigation, Methodology, Software, Validation, Writing – review & editing. SO: Data curation, Formal analysis, Investigation, Methodology, Software, Validation, Writing – review & editing. HN: Data curation, Formal analysis, Investigation, Methodology, Validation, Writing – review & editing. LA: Data curation, Formal analysis, Investigation, Methodology, Validation, Writing – review & editing. OC: Formal analysis, Investigation, Methodology, Writing – review & editing, Data curation, Software. BC: Data curation, Formal analysis, Investigation, Methodology, Validation, Writing – review & editing. XL: Investigation, Resources, Supervision, Validation, Writing – review & editing. ST-A: Conceptualization, Formal analysis, Funding acquisition, Investigation, Methodology, Project administration, Resources, Supervision, Validation, Visualization, Writing – original draft, Writing – review & editing.
