# Supplementary figures and images for: Metal tolerance and biosorption capacities of bacterial strains isolated from an urban watershed
Source: Front Microbiol. 2023 Oct 23;14:1278886. doi: 10.3389/fmicb.2023.1278886 (PMC10630031; doi:10.3389/fmicb.2023.1278886)

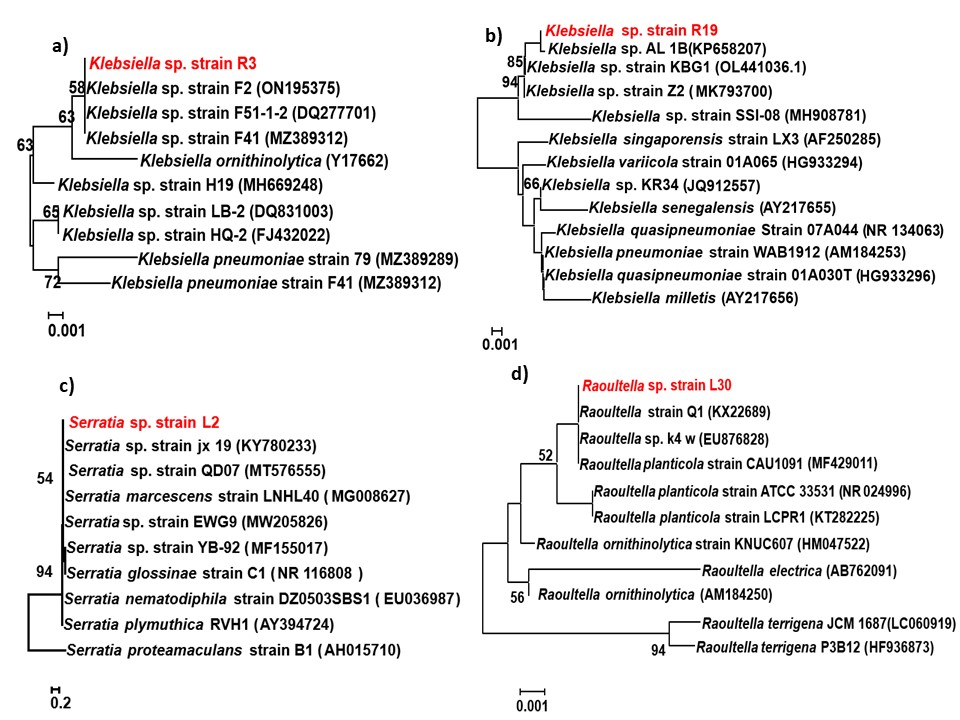

Supplement: Supplementary file 1 [file Image_1.JPEG]

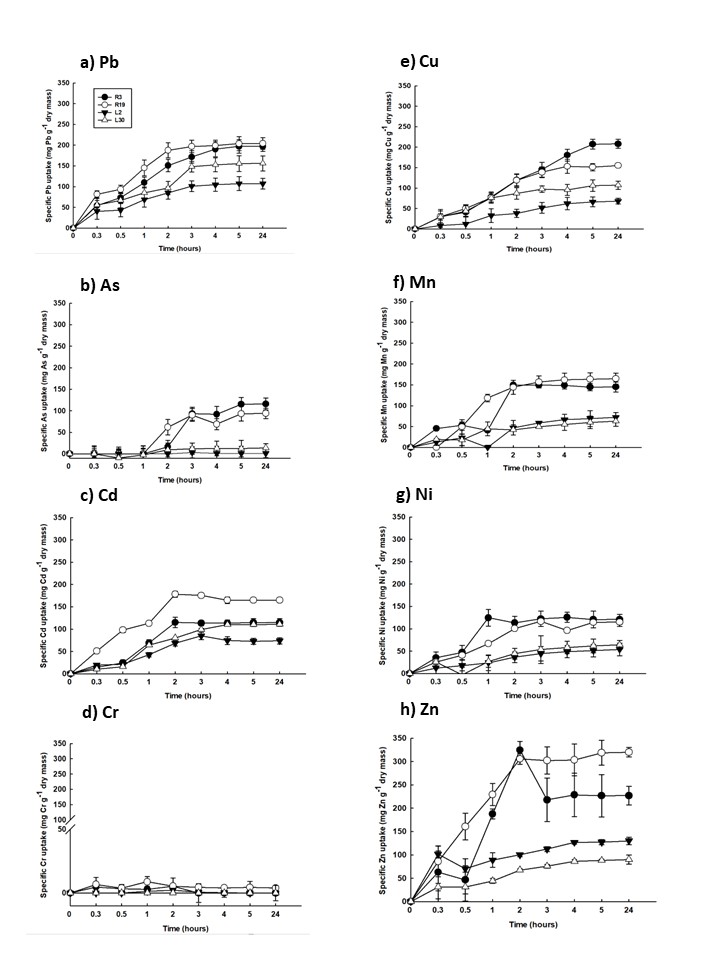

Supplement: Supplementary file 2 [file Image_2.JPEG]

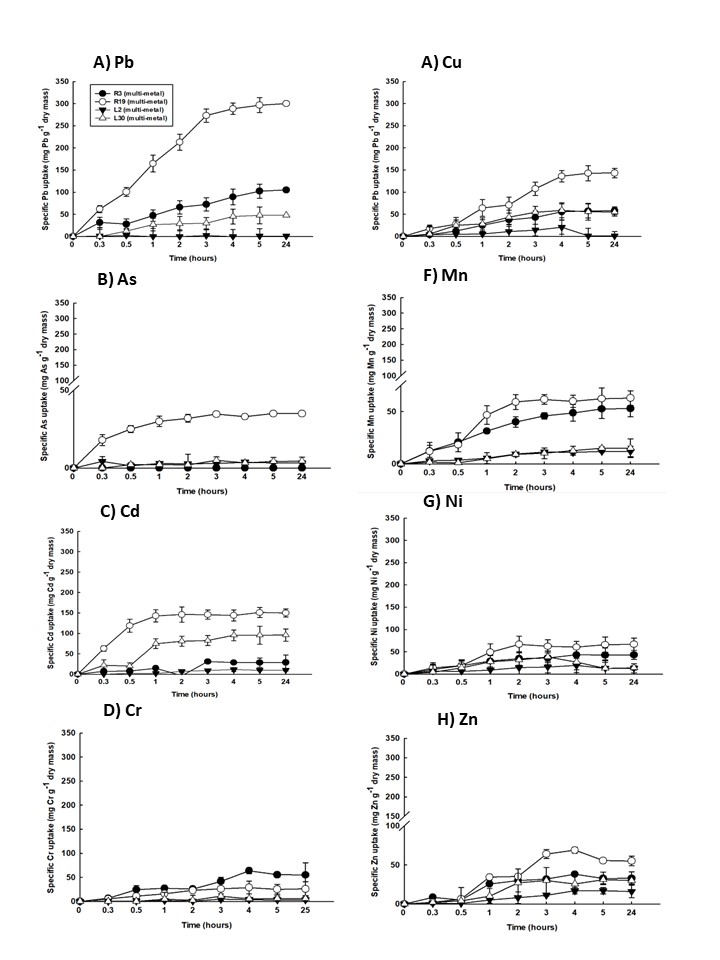

Supplement: Supplementary file 3 [file Image_3.JPEG]
