# Supplementary material for: Metal tolerance and biosorption capacities of bacterial strains isolated from an urban watershed
Source: Front Microbiol. 2023 Oct 23;14:1278886. doi: 10.3389/fmicb.2023.1278886 (PMC10630031; doi:10.3389/fmicb.2023.1278886)
Supplement: Supplementary file 4 [file Table_1.DOCX]

**Table 1** Minimum inhibitory concentrations of metals.

| Minimum inhibitory  concentrations (MIC)  (mg L^-1^) | Strains | | | |
| --- | --- | --- | --- | --- |
|  | *Klebsiella* sp. strain R3 | *Klebsiella* sp. strain R19 | *Serratia* sp.  strain L2 | *Raoultella* sp. strain L30 |
|  |  |  |  |  |
| As | 250 | 350 | 350 | 450 |
| Cd | 20 | 20 | 75 | 75 |
| Cr | 10 | 15 | 15 | 15 |
| Cu | 50 | 50 | 20 | 50 |
| Pb | 700 | 800 | 800 | 800 |
| Mn | >2000 | >2000 | >2000 | >2000 |
| Ni | 50 | 50 | 75 | 50 |
| Zn | 1000 | 500 | 1100 | 1100 |
|  |  |  |  |  |
