# Supplementary material for: Metal tolerance and biosorption capacities of bacterial strains isolated from an urban watershed
Source: Front Microbiol. 2023 Oct 23;14:1278886. doi: 10.3389/fmicb.2023.1278886 (PMC10630031; doi:10.3389/fmicb.2023.1278886)
Supplement: Supplementary file 5 [file Table_2.DOCX]

**Table 2** IR absorption band changes and possible assignment for the metal-free and metal-loaded *Klebsiella* strain sp. R3.

| FTIR peak |  |  | *Klebsiella* sp. strain R3 | | |  | |
| --- | --- | --- | --- | --- | --- | --- | --- |
|  | metal- free | metal -loaded | | Displace-ment* | Functional groups | | Bond Assignment |
|  |  |  | |  |  | |  |
| 1  2  3  4  5  6  7  8  9  10  11  12  13  14  15  16  17  18  19  20  21  22  23  24  25  26  27  28  29 | 719  738  805  1457  1558  1647  2121  2147  2177  2195  2251  2859  2986  3630 | 667  712  742  798  846  876  1003  1128  1282  1469  1557  1640  1700  1718  1770  2117  2136  2162  2184  2199  2259  2861  2984  3120  3224  3280  3493  3993  3623 | | 667  7  4  7  846  876  1003  1128  1282  12  1  7  1700  1718  1770  4  11  2162  7  4  8  3  2  3120  3224  3280  3493  3593  7 | C_2_H_2_R_2_  1,3-Disubstituted (Aromatic compounds)  1,2-Disubstituted (Aromatic compounds)  C_2_HR_3_  1,4-Disubstituted (Aromatic compounds)  1,3-Disubstituted (Aromatic compounds)  (RCO)_2_O  R-OH  RCOOR’  C-C  P-NH_2_  R_2_C=NR or R_2_C=NH  R_2_C=O or RCOOH  R_2_C=O or RCOOH  RCOC1  C≡C  C≡C  C≡C  C≡C  C≡C  RC≡N  C-H  P-NH  C=C-H  P-NH_2_  P-NH_2_  RO-H hydrogen bond  RO-H free  RO-H free | | C-H out-of-plane-bend Alkene  C-H out-of-plane-bends Aromatic  C-H out-of-plane-bend Aromatic  C-H out-of-plane-bend Alkene  C-H out-of-plane-bend Aromatic  C-H out-of-plane-bend Aromatic  C-O stretch Carbonyl  C-O stretches Alcohol  C-O stretch Carbonyl  C-C bend Alkane  NH_2_ Amine  C=N stretch Imine and Oxime  C=O stretch Ketone or carboxylic acid  C=O stretch Ketone or carboxylic acid  C=O stretch Acid Chloride  C≡C stretch Alkyne  C≡C stretch Alkyne  C≡C stretch Alkyne  C≡C stretch Alkyne  C≡C stretch Alkyne  C≡N stretch Nitrile  C-H stretch Alkane  NH Amine  C-H stretch Alkene  NH_2_ Amine  NH_2_ Amine  O-H stretch Hydroxyl  O-H stretch Hydroxyl  O-H stretch Hydroxyl |

*IR band shifts in red; new bands in blue
