# Supplementary material for: Metal tolerance and biosorption capacities of bacterial strains isolated from an urban watershed
Source: Front Microbiol. 2023 Oct 23;14:1278886. doi: 10.3389/fmicb.2023.1278886 (PMC10630031; doi:10.3389/fmicb.2023.1278886)
Supplement: Supplementary file 6 [file Table_3.DOCX]

**Table 3** IR absorption band changes and possible assignment for the metal-free and metal-loaded *Klebsiella* sp. strain R19.

| FTIR peak |  | | |  | *Klebsiella* sp. strain R19 | | | | |  | |
| --- | --- | --- | --- | --- | --- | --- | --- | --- | --- | --- | --- |
|  | metal- free |  | metal- loaded | | |  | Displace-ment* | Functional groups | Bond Assignment | |  |
|  |  |  |  | | |  |  |  |  | |  |
| 1  2  3  4  5  6  7  8  9  10  11  12  13  14  15  16  17  18  19  20  21  22  23  24  25  26  27  28  29  30 | 716  734  801  962  1230  1368  1390  1435  1457  1487  2166  2188    2837  2881  2926 |  | 675  719  737  768  813  846  924  939  964  988  1118  1238  1373  1393  1434  1461  1490  2147  2162  2193  2210  2230  2232  2840  2889  2911  2930  2960  2986  2997 | | |  | 675  3  4  768  12  846  924  939  2  988  1118  8  5  3  1  4  2  2147  4  5  2210  2230  2232  3  8  2911  2930  34  2986  2997 | C_2_H_2_R_2_  1,3-Disubstituted (Aromatic compounds)  Monosubstituted  1,2-Disubstituted (Aromatic compounds)  C_2_HR_3_  1,4-Disubstituted (Aromatic compounds)  P-NH_2_  P-NH_2_  C_2_H_2_R_2_  C_2_H_3_R  R-OH  R-OH  R-NO_2_  R-NO_2_  C-C  C-C  C=C  C≡C  C≡C  C≡C  C≡C  C≡C  C≡C  RCHO  C-H  C-H  C-H  C-H  P-NH  P-NH | C-H out-of-plane bend Alkene  C-H out-of-plane bend Aromatic  C-H out-of-plane bend Aromatic  C-H out-of-plane bend Aromatic  C-H out-of-plane-bend Alkene  C-H out-of-plane-bend Aromatic  NH_2_ Amine  NH_2_ Amine  C-H out-of-plane-bend Alkene  C-H out-of-plane-bends Alkene  C-O stretches Alcohol  C-O stretches Alcohol  N=O Nitro  N=O Nitro  C-C bend Alkane  C-C bend Alkane  C=C Aromatic  C≡C stretch Alkyne  C≡C stretch Alkyne  C≡C stretch Alkyne  C≡C stretch Alkyne  C≡C stretch Alkyne  C≡C stretch Alkyne  C-H stretch Aldehyde  C-H stretch Alkane  C-H stretch Alkane  C-H stretch Alkane  C-H stretch Alkane  NH Amine  NH Amine | |  |

*IR band shifts in red; new bands in blue
