# Supplementary material for: Metal tolerance and biosorption capacities of bacterial strains isolated from an urban watershed
Source: Front Microbiol. 2023 Oct 23;14:1278886. doi: 10.3389/fmicb.2023.1278886 (PMC10630031; doi:10.3389/fmicb.2023.1278886)
Supplement: Supplementary file 7 [file Table_4.DOCX]

**Table 4** IR absorption band changes and possible assignment for the metal-free and metal-loaded *Serratia* sp. strain L2.

| FTIR peak |  | |  | | *Serratia* sp. strain L2 | | | | |  | |
| --- | --- | --- | --- | --- | --- | --- | --- | --- | --- | --- | --- |
|  | metal- free |  | | metal-  loaded | |  | Displace- ment | Functional groups | Bond Bond Assignment | |  |
|  |  |  | |  | |  |  |  |  | |  |
| 1  2  3  4  5  6  7  8  9  10  11  12  13  14  15  16  17  18  19 | 719  2132  2154  2195  2218  2851  2922  2956 |  | | 663  704  723  846  909  1092  1435  1469  2134  2158  2184  2214  2837  2885  2930  2960  2986  3496  3623 | |  | 663  704  4  846  909  1092  1435  1469  4  4  11  4  2837  34  8  4  2986  3496  3623 | P-NH  Monosubstituted (Aromatic Compound)  1,3-Disubstituted (Aromatic Compound)  1,4-Disubstituted (Aromatic Compound)  C_2_H_3_R  R-OH  C-C  C-C  C≡C  C≡C  C≡C  C≡C  RCHO  C-H  C-H  C-H  P-NH  RO-H hydrogen bond  RO-H free | NH_2_ Amine  C-H out-of-plane-bends Aromatic  C-H out-of-plane-bend Aromatic  C-H out-of-plane-bend Aromatic  C-H out-of-plane-bends Alkene  C-O stretches Alcohol  C-C bend Alkane  C-C bend Alkane  C≡C stretch Alkyne  C≡C stretch Alkyne  C≡C stretch Alkyne  C≡C stretch Alkyne  C-H stretch Aldehyde  C-H stretch Alkane  C-H stretch Alkane  C-H stretch Alkane  NH Amine  O-H stretch Hydroxyl  O-H stretch Hydroxyl | |  |

*IR band shifts in red; new bands in blue
