# Supplementary material for: Metal tolerance and biosorption capacities of bacterial strains isolated from an urban watershed
Source: Front Microbiol. 2023 Oct 23;14:1278886. doi: 10.3389/fmicb.2023.1278886 (PMC10630031; doi:10.3389/fmicb.2023.1278886)
Supplement: Supplementary file 8 [file Table_5.DOCX]

**Table 5** IR absorption band changes and possible assignment for the metal-free and metal-loaded *Raoultella* sp. strain L30 strain.

| FTIR peak |  | |  | | *Raoultella* sp. strain L30 | | | | |  | |
| --- | --- | --- | --- | --- | --- | --- | --- | --- | --- | --- | --- |
|  | metal- free |  | | metal- loaded | |  | Displace-ment | Functional groups | Bond Assignment | |  |
|  |  |  | |  | |  |  |  |  | |  |
| 1  2  3  4  5  6  7  8  9  10  11  12  13  14  15  16  17  18  19  20  21  22 | 734  805  846  887  1461  2188  2855  2922 |  | | 678  738  809  848  886  1066  1260  1465  1562  1640  2147  2162  2190  2210  2230  2840  2859  2930  2986  2997  3493  3586 | |  | 678  4  4  2  1  1066  1260  4  1562  1640  2147  2162  2  2210  2230  2840  4  8  2986  2996  3493  3586 | C_2_H_2_R_2_  Monosubstituted (Aromatic Compound)  C_2_HR_3_  1,4-Disubstituted (Aromatic Compound)  C_2_H_2_R_2_  (RCO)_2_O  R-OH  C-C  P-NH_2_  R_2_C=NR or R_2_C=NH  C≡C  C≡C  C≡C  C≡C  C≡C  RCHO  C-H  C-H  P-NH  P-NH  RO-H hydrogen bond  RO-H free | C-H out-of-plane bend Alkene  C-H out-of-plane bends Aromatic  C-H out-of-plane bends Alkene  C-H out-of-plane bends Aromatic  C-H out-of-plane bend Alkene  C-O stretch Carbonyl  C-O stretches Alcohol  C-C bend Alkane  NH_2_ Amine  C=N stretch Imine and Oxime  C≡C stretch Alkyne  C≡C stretch Alkyne  C≡C stretch Alkyne  C≡C stretch Alkyne  C≡C stretch Alkyne  C-H stretch Aldehyde  C-H stretch Alkane  C-H stretch Alkane  NH Amine  NH Amine  O-H stretch Hydroxyl  O-H stretch Hydroxyl | |  |

*IR band shifts in red; new bands in blue
