# Supplementary material for: Metal tolerance and biosorption capacities of bacterial strains isolated from an urban watershed
Source: Front Microbiol. 2023 Oct 23;14:1278886. doi: 10.3389/fmicb.2023.1278886 (PMC10630031; doi:10.3389/fmicb.2023.1278886)
Supplement: Supplementary file 9 [file Table_6.DOCX]

**Table S1** Morphological and biochemical characteristics of the bacterial isolates.

|  | Bacterial strains | | | |
| --- | --- | --- | --- | --- |
| Characteristics | *Klebsiella* sp. strain R3 | *Klebsiella* sp. strain R19 | *Serratia* sp.  strain L2 | *Raoultella* sp. strain L30 |
| Colony color | cream | cream | cream | cream |
| Colony morphology |  | circular | circular | circular |
| Appearance | Circular, smooth shiny | Circular, smooth shiny | Circular, smooth shiny | Circular, smooth shiny |
| Elevation | Convex | Convex | Convex | Convex |
| Margin | Entire | Entire | Entire | Entire |
| Gram reaction | Gram negative | Gram negative | Gram negative | Gram negative |
| Cell morphology | rod | rod | rod | rod |
| Motility  Slime/EPS production | non-motile  positive | non-motile  positive | motile  negative | motile  positive |
|  |  |  |  |  |
| *Growth* |  |  |  |  |
| Temperature |  |  |  |  |
| 4^o^C | - | - | - | - |
| 20^o^C | + | + | + | + |
| 37^o^C | + | + | + | + |
| 40^o^C | + | + | + | + |
| 50^o^C | + | + | + | + |
| Aerobic growth | + | + | + | + |
| Anaerobic growth | + | + | + | + |
|  |  |  |  |  |
| *Biochemical reactions* |  |  |  |  |
| Oxidase | - | - | + | - |
| Catalase | + | + | + | + |
| β-galactosidase | + | + | + | + |
| Arginine dihydrolase | + | - | - | - |
| Lysine decarboxylase | + | + | + | + |
| Ornithine decarboxylase | + | - | + | - |
| Citrate utilization | + | + | + | + |
| Sulfide production | - | - | - | - |
| Urease | + | + | + | + |
| Tryptophan deaminase | - | - | + | + |
| Indole production | + | + | + | + |
| Vogues-Proskauer reaction | + | - | + | + |
| Gel liquefaction | + | - | + | - |
| Glucose fermentation | - | + | - | - |
| Mannitol fermentation | - | + | - | - |
| Nitrate reduction | + | + | + | + |
| Sorbitol fermentation | + | + | + | + |
| Rhamnose fermentation | + | + | + | + |
| Sucrose fermentation | + | + | + | + |
| Melibiose fermentation | + | + | + | + |
| Amygdalin fermentation | + | + | + | + |
| Arabinose fermentation | + | + | + | + |
