# Supplementary material for: Metal tolerance and biosorption capacities of bacterial strains isolated from an urban watershed
Source: Front Microbiol. 2023 Oct 23;14:1278886. doi: 10.3389/fmicb.2023.1278886 (PMC10630031; doi:10.3389/fmicb.2023.1278886)
Supplement: Supplementary file 10 [file Table_7.DOCX]

**Supplementary materials: (Figure legends)**

**Fig. S1.** Neighbor-joining trees showing the phylogenetic positions of the bacterial strains a) *Klebsiella* sp*.* strain R3, b) *Klebsiella* sp. strain R19, c) *Serratia* sp. strain L2, and d) *Raoultella* sp. strain L30 identified based on 16S rRNA sequences. The numbers at the nodes include the level of bootstrap on neighbor-joining analysis of 1,000 replicates. Accession numbers of references are given in parenthesis.

**Fig. S2.** Metal removal in single metal solutions by the four bacterial strains at different time interval. All the values are mean of three replicates ± standard deviation.

**Fig. S3.** Metal removal in multi-metal solutions by the four bacterial strains at different time intervals. All the values are mean of three replicates ± standard deviation.
